# Nucleolar stress in *Drosophila* neuroblasts, a model for human ribosomopathies

**DOI:** 10.1242/bio.046565

**Published:** 2020-04-28

**Authors:** Sonu Shrestha Baral, Molly E. Lieux, Patrick J. DiMario

**Affiliations:** Department of Biological Sciences, Louisiana State University, Baton Rouge, LA 70803, USA

**Keywords:** Nucleolar stress, Neuroblasts, *Drosophila*, Nopp140, Ribosomopathy

## Abstract

Different stem cells or progenitor cells display variable threshold requirements for functional ribosomes. This is particularly true for several human ribosomopathies in which select embryonic neural crest cells or adult bone marrow stem cells, but not others, show lethality due to failures in ribosome biogenesis or function (now known as nucleolar stress). To determine if various *Drosophila* neuroblasts display differential sensitivities to nucleolar stress, we used CRISPR-Cas9 to disrupt the *Nopp140* gene that encodes two splice variant ribosome biogenesis factors (RBFs). Disruption of *Nopp140* induced nucleolar stress that arrested larvae in the second instar stage. While the majority of larval neuroblasts arrested development, the mushroom body (MB) neuroblasts continued to proliferate as shown by their maintenance of deadpan, a neuroblast-specific transcription factor, and by their continued EdU incorporation. MB neuroblasts in wild-type larvae appeared to contain more fibrillarin and Nopp140 in their nucleoli as compared to other neuroblasts, indicating that MB neuroblasts stockpile RBFs as they proliferate in late embryogenesis while other neuroblasts normally enter quiescence. A greater abundance of Nopp140 encoded by maternal transcripts in *Nopp140-/-* MB neuroblasts of 1­­­–2-day-old larvae likely rendered these cells more resilient to nucleolar stress.

## Abstract (precis)

**Summary:** Nucleolar stress (loss of ribosome production/function) in certain human progenitor or stem cells results in disease. In fruit flies, larval mushroom body neuroblasts are relatively resilient to nucleolar stress.

## INTRODUCTION

The nucleolus is the nuclear sub-compartment responsible for ribosomal subunit biogenesis (Baßler and Hurt, 2019). Functional ribosomes in the cytoplasm of eukaryotic cells consist of the small ribosomal subunit with its 18S ribosomal RNA (rRNA) assembled with 33 ribosomal proteins and the large ribosomal subunit with its 28S, 5.8S, and 5S rRNAs assembled with 47 ribosomal proteins. Subunit assembly is a complex choreography of reactions and interactions within nucleoli beginning with pre-rRNA transcription by RNA polymerase I (RNA Pol I) on tandemly repeated ribosomal DNA (rDNA) genes. The 38S pre-rRNA in *Drosophila* undergoes endonuclease cleavages to generate 18S, 5.8S+2S, and 28S rRNAs (Long and Dawid, 1980). These rRNAs undergo 2′-*O*-methylation by box C/D small nucleolar ribonucleoprotein complexes (snoRNPs) and pseudouridylation by box H/ACA snoRNPs (Wang et al., 2002; Yang et al., 2000; Bachellerie et al., 2002). Besides endonucleases and snoRNPs, subunit biogenesis requires a myriad of other factors serving as RNP chaperones, RNA helicases, and GTPase release factors (Kressler et al., 2010). The chaperones, often referred to as ribosome biogenesis factors (RBFs), act early in ribosome assembly; they include Nopp140 (Nucleolar and Cajal body phosphoprotein of 140 kDa) and treacle. While both Nopp140 and treacle are found in vertebrates, only Nopp140 orthologues are expressed in all eukaryotes.

Ribosome biogenesis requires high-energy expenditures by the cell; approximately 60% of total cellular transcription is devoted to rRNA, with some 2000 ribosomes assembled per minute in actively growing yeast cells (Warner, 1999; Woolford and Baserga, 2013). Any perturbation in ribosome biogenesis disrupts cell homeostasis; this is now called nucleolar (or ribosome) stress (Golomb et al., 2014; Tsai and Pederson, 2014; Yang et al., 2018). In humans, nucleolar stress due to mutations in RBFs, processing snoRNPs, or the ribosomal proteins themselves results in disease states collectively called ribosomopathies, of which there are several (Narla and Ebert, 2010). While each ribosomopathy has its own distinct phenotypes, and several display tissue-specificity (McCann and Baserga, 2013), there are commonalities among them: the most prevalent dysfunctions include craniofacial abnormalities, other skeletal defects, and bone marrow failures. All ribosomopathies affect only certain stem cells or progenitors despite the mutation being systemic.

One of these ribosomopathies is the Treacher Collins Syndrome (TCS), a congenital set of craniofacial birth defects caused by haplo-insufficiency mutations in the *TCOF1* gene that encodes treacle (Sakai and Trainor, 2009). In TCS individuals, a particular subset of neural crest cells that normally migrate to and populate pharyngeal arches I and II on day 24–25 of human embryogenesis is insufficient in functional ribosomes. This leads to p53-dependent apoptosis (Jones et al., 2008), and loss of these select neural crest cells causes the craniofacial defects. A TCS-like phenotype can also result from mutations in genes encoding RNA Pol I and III subunit proteins, POLR1D and POLR1C, respectively (Dauwerse et al., 2011; Noack Watt et al., 2016). The question is, why are only certain progenitor cells affected while many others remain resilient?

Like treacle, metazoan Nopp140 orthologues contain alternating acidic and basic motifs constituting a large central domain of low sequence complexity (Meier, 1996). Treacle and Nopp140 also share similar roles in chaperoning C/D-box snoRNPs to the dense fibrillar component of nucleoli where pre-rRNA is modified by site-specific 2′-*O*-methylation. Unlike treacle, Nopp140 locates to Cajal bodies; thus Nopp140 may also play a role in snoRNP assembly and transport to nucleoli (Gonzales et al., 2005; Hayano et al., 2003; He et al., 2015). While a length polymorphism exists for the *Drosophila Nopp140* gene (Baral and DiMario, 2019), and a mutation in the orthologous *Caenorhabditis* *elegans* Nopp140 gene, *dao-5*, has been described (Lee et al., 2014), no mutations in the human Nopp140 gene, *NOLC1*, have been characterized, suggesting a *de novo* mutation in *NOLC1* may be embryonic lethal.

With predictably critical roles in ribosome biogenesis, we used RNAi to deplete Nopp140 in *Drosophila* (Cui and DiMario, 2007) to generate *Minute*-like phenotypes (Lambertsson, 1998; Marygold et al., 2007). Resulting nucleolar stress studies showed cell death occurred either by apoptosis in imaginal disc cells or by autophagy in terminally differentiated polyploid gut cells; these stress responses were p53-independent, but JNK-dependent (James et al., 2013). We used *pBac* elements to knock-out the *Drosophila Nopp140* gene, and showed a tremendous loss of cytoplasmic ribosomes in larval polyploid cells, a corresponding accumulation of unusual electron dense granules in the cytoplasm of these same cells, a redistribution of fibrillarin from the nucleoli to the nucleoplasm in several cell types, and a resulting overall reduction in 2′-*O*-methylation of pre-rRNA (He et al., 2015). We did not see a detectable loss in pre-rRNA synthesis or gross morphological changes in nucleolar structure (He et al., 2015). We are now testing the hypothesis that defective ribosomes are still assembled in the absence of Nopp140, but then quickly degraded in the cytoplasm.

**Fig. 1.**
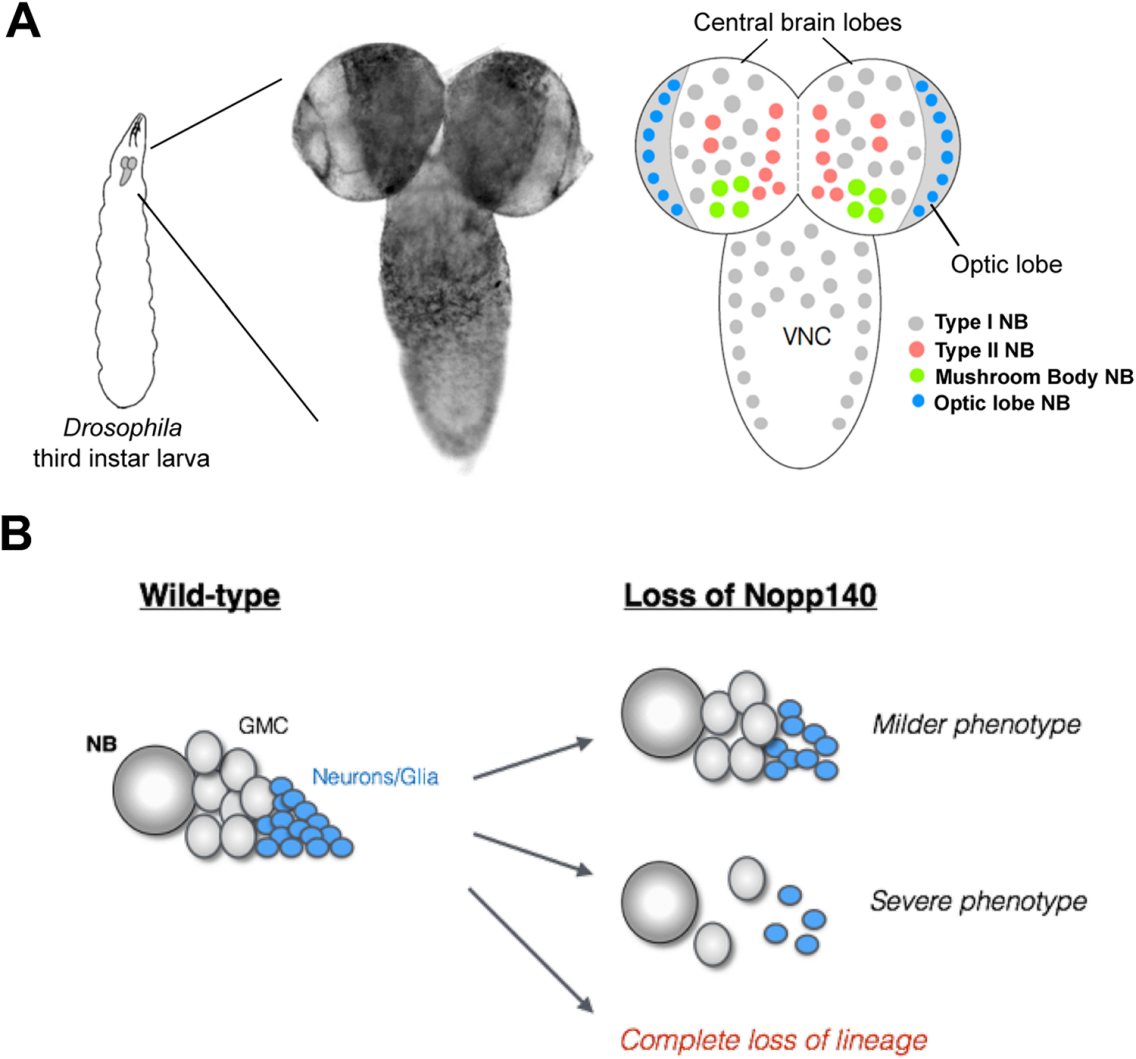
**Anatomy of the *Drosophila* larval brain and overall hypothesis.** (A) Larval brains have two central brain lobes and a ventral nerve cord (VNC). There are roughly four NB types within the larval brain: Type I (grey), Type II (red), MB (green), and optic lobe (blue). These NBs are shown in their putative locations within the larval brain. MB NBs reside in the posterior of the brain lobes, which often flip forward when placing the brain on a microscope slide, thus giving a false impression of an anterior location within the brain lobe. (B) Our hypothesis is that upon nucleolar stress due to loss of Nopp140, different NB lineages exhibit variable phenotypes ranging from a mild to severe loss of lineage progeny cells, to compete loss of the lineage altogether.

To investigate the underlying mechanisms contributing to stem cell or progenitor cell specificity as seen in the human ribosomopathies, we initiated a study of nucleolar stress in *Drosophila* larval neuroblasts. We wanted to determine if all neuroblast types respond similarly or differentially to nucleolar stress. The *Drosophila* larval brain comprises distinct neuroblast (NB) lineages generated from a fixed set of founder NBs (Homem and Knoblich, 2012; Hartenstein and Wodarz, 2013). Briefly, there are four major NB types in the *Drosophila* larval brain; Type I, Type II, mushroom body (MB), and optic lobe NBs (Fig. 1A). We initially hypothesized that upon nucleolar stress caused by the loss of Nopp140, different NB lineages exhibit variable phenotypes ranging from a mild loss of lineage progeny cells to substantial loss of the lineage altogether (Fig. 1B). Here we show that MB NBs are more resilient to the effects of nucleolar stress compared to the other NB types. Hence, different NB lineages respond variably to nucleolar stress, which is reminiscent of the neural crest cell-specific effects caused by the loss of treacle in TCS individuals.

## RESULTS

### CRISPR for homology directed repair (HDR) to disrupt the *Nopp140* gene

**Fig. 2.**
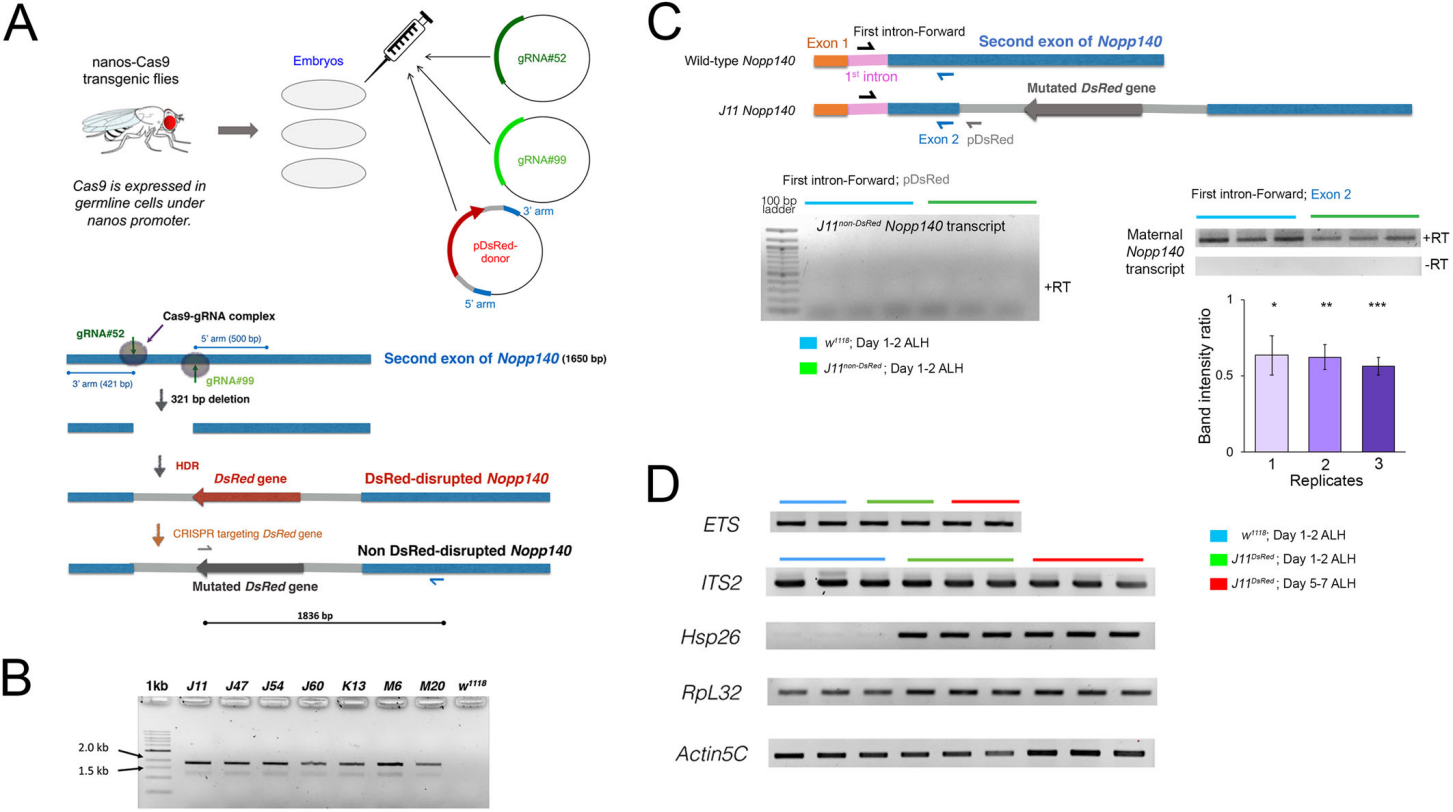
**CRISPR-mediated disruption of the *Nopp140* gene and RT-PCR analyses.** (A) Three plasmids encoding guide RNAs, gRNA#52 and gRNA#99, or the DsRed protein were injected into embryos from the *nanos-Cas9* fly line. The guide RNAs directed Cas9 cleavage at two specific sites located 321 base pairs apart in the second exon of the *Nopp140* gene (blue bar; 1650 bp total). The *DsRed* gene (red arrow) with flanking plasmid sequences (light grey) and 3′ and 5′ *Nopp140* homology arms were inserted into the deletion by HDR. Seven heterozygous *Nopp140* disruption lines were identified by DsRed expression in adult eyes. The *DsRed* gene was subsequently mutated (dark grey arrow) by CRISPR-mediated mutagenesis. (B) Genomic PCRs using a *DsRed*-specific forward primer (grey half arrow in panel A) and a downstream *Nopp140*-specific reverse primer (blue half arrow in panel A) verified the *DsRed* insertion within the second exon of *Nopp140* gene in all seven *Nopp140* heterozygous disruption lines (*J11*, *J47*, *J54*, *J60*, *K13*, *M6* and *M20*) with the expected 1836 bp product. The *w^1118^* fly line served as negative control. (C) RT-PCR analyses of *Nopp140* transcript levels in control *w^1118^* and homozygous *J11^non-DsRed^* larvae day 1–2 ALH using the Exon2 reverse primer (blue half arrow) for first strand cDNA synthesis. Subsequent PCRs used the same Exon2 reverse primer and a forward primer specific for the first intron of the *Nopp140* gene. A representative gel is provided. No PCR products appeared in the minus-RT controls. Band intensity ratios (*J11/w^1118^*) were determined for three biological replicates with overall mean±s.e.m. of 0.61±0.022. Student's *t*-test: two-tailed with equal variance for three biological replicates (1, 2, 3) with three PCR technical replicates each, *P*-values=0.037*, 0.00081**, 0.0064***, respectively. No RT-PCR product was detected using the pDsRed reverse primer (grey half arrow) in the *J11^non-DsRed^* disruption line. (D) RT-PCR analyses of *ETS*, *ITS2*, *Hsp26*, *RpL32* and *Actin5C* transcript levels were carried out in control *w^1118^* larvae at day 1–2 ALH and in homozygous *J11^DsRed^* larvae at two time points, day 1–2 and 5–7 ALH using gene-specific reverse primers. Three biological replicates were performed. For each replicate, total RNA was extracted from ∼300 *w^1118^* and 150–300 homozygous *J11* larvae. PCR reactions were carried out in triplicate for each first strand cDNA. Representative gels are shown.

We used CRISPR-Cas9 to delete a target sequence of 321 bps from the second exon of the *Nopp140* gene*.* A cocktail of two *gRNA* plasmids and the *DsRed-Donor* plasmid was injected into embryos homozygous for the *nanos*::*Cas9* transgene (Fig. 2A). The gRNAs directed the Cas9-mediated deletion, and HDR inserted the *DsRed* gene across the deletion (Fig. 2A). *DsRed* then served as a selectable marker for the disrupted *Nopp140* gene; it was expressed from the *3xP3* eye promoter, which is normally active in the entire embryonic and larval brain, Bolwig's organ, hind gut, anal pads and adult eyes. We recovered seven independent *Nopp140* disruption lines (*J11*, *J47*, *J54*, *J60*, *K13*, *M6* and *M20*) using the red fluorescence eye phenotype. Each of the seven *Nopp140*-disrupted chromosomes was maintained over the *TM3-GFP* balancer chromosome that carries a wild-type copy of *Nopp140*. The *DsRed* insertion was verified by genomic PCRs (Fig. 2B). The expected 1836 bp PCR product was amplified in all seven *Nopp140* insertion alleles, with *w^1118^* acting as a wild-type negative control (Fig. 2B). Among the seven lines initially recovered, *J11^DsRed^/TM3-GFP* was backcrossed with the *Sb^1^*/*TM3-GFP* fly line for at least six generations to eliminate possible off-target mutations in the *J11^DsRed^* line.

The *GFP* reporter gene on the *TM3* balancer chromosome is expressed in a small cluster of larval midgut cells that are easily identifiable. Therefore, with *inter se* crosses of *J11^DsRed^/TM3-GFP* stock flies, we hand-selected larvae that were homozygous for *J11^DsRed^* versus larvae that were heterozygous for *J11^DsRed^/TM3-GFP* with prominent GFP signals in their midgut.

To conduct multi-channel immuno-fluorescence of the *Drosophila* brain, we again used CRISPR-Cas9, but now with non-homologous end joining (NHEJ) to disrupt the *DsRed* gene inserted in the *J11^DsRed^* allele. A cocktail of two gRNA plasmids and the *pBS-Hsp70-Cas9* vector injected into *J11^DsRed^/TM3* embryos produced several independent fly lines with mutations in *DsRed* (Fig. S1). We sequenced the second exon region in two of these lines, *A5* and *A7*, and verified that each had a short deletion at the gRNA#3 target site within the *DsRed* gene (Fig. S1). The *A7-J11^non-DsRed^* fly line was again backcrossed six times to deplete any possible off-site targets. Either the original *J11^DsRed^* fly line or its derived A7*-J11^non-DsRed^* line was used for the experiments described below.

We next performed RT-PCR analyses to test if the disrupted *Nopp140* gene was transcribed in homozygous *A7*-*J11^non-DsRed^* larvae (day 1–2 ALH) (Fig. 2C). The reverse primer referred to as *pDsRed* in Fig. 2C annealed to the *pDsRed-attP* plasmid sequence a few base pairs downstream of the junction between the *Nopp140* second exon and the *DsRed* donor sequence. No transcripts containing *DsRed*-*att* sequences were detected in the total RNA samples prepared from homozygous *A7-J11^non-DsRed^* larvae 1–2 day ALH, similar to the *w^1118^* sample that served as a negative control (Fig. 2C, but see below for a positive control). Nonsense-mediated decay (NMD) would likely degrade *Nopp140* pre-mRNAs transcribed from the disrupted gene as they would likely contain premature stop codons within the *pDsRed-att* sequences, or these pre-mRNAs may be improperly/incompletely spliced (Garneau et al., 2007). This lack of RT-PCR products eliminates the likelihood of a dominant-negative effect due to the production of truncated Nopp140 proteins encoded by the disrupted *Nopp140* gene*.* In summary, hand-selected larvae homozygous for the *Nopp140 J11^non-DsRed^* allele provide a null genotype (*Nopp140−/−*) systemic throughout the larvae.

We then determined if there were maternal wild-type *Nopp140* transcripts present in the RNA preparations isolated from the same homozygous *A7-J11^non-DsRed^* 1–2 day ALH larvae used for the same RT-PCRs described in the preceding section. We performed these second RT reactions using a reverse primer (*Exon2*, blue in Fig. 2C) that anneals to the *Nopp140* second exon a few base pairs upstream of the junction between the *Nopp140* second exon and the *DsRed* donor sequence. Since *Nopp140* transcripts harboring *DsRed* sequences were undetectable in these same larvae, first strand cDNAs primed with *Exon2* should indicate the presence of maternal *Nopp140* transcripts in homozygous *J11^DsRed^* larvae, and thus serve as a positive control for the initial RT-PCRs that showed an absence of *DsRed-att*-containing transcripts. These second RT-PCRs shown on the right in Fig. 2C indicated that maternal *Nopp140* transcripts were indeed present in the *Nopp140−/−* larvae at day 1–2 ALH. The bar graph in Fig. 2C indicates that the abundance of maternal *Nopp140* transcripts in the *Nopp140−/−* larvae was about half that seen in wild-type larvae, suggesting that both maternal and zygotic *Nopp140* transcript pools exist in early wild-type larvae (Fig. 2C).

As additional controls (Fig. 2D), RT-PCRs of the external transcribed spacer (*ETS*) and the internal transcribed spacer 2 (*ITS2*) sequences within pre-rRNA showed that their levels were unaffected in homozygous *J11^DsRed^* larvae at day 1–2 ALH and at day 5–7 ALH. This indicates that loss of Nopp140 had no effect on rDNA transcription, which agrees with our earlier observations on the *pBac*-generated *Nopp140^KO121^* deletion (He et al., 2015). Furthermore, *Hsp26* transcript levels were upregulated in homozygous *J11^DsRed^* larvae at both day 1–2 and day 5–7 ALH, whereas the wild-type larvae had almost undetectable levels of *Hsp26* transcript (Fig. 2D). Overexpression of *Hsp26* in homozygous *J11^DsRed^* larvae as early as day 1 ALH indicated a cellular stress response due to the effects of Nopp140 loss (e.g. Wang et al., 2004). As final controls, we accessed *RpL32* and *Actin5C* transcript levels: while *RpL32* transcript levels remained unchanged between the wild-type and homozygous *J11^DsRed^* samples and between biological replicates, *Actin5C* transcript levels fluctuated slightly within the samples and between biological replicates for reasons that remain uncertain.

### Maternal Nopp140 protein is reduced in early *J11 Nopp140−/−* larval brains

**Fig. 3.**
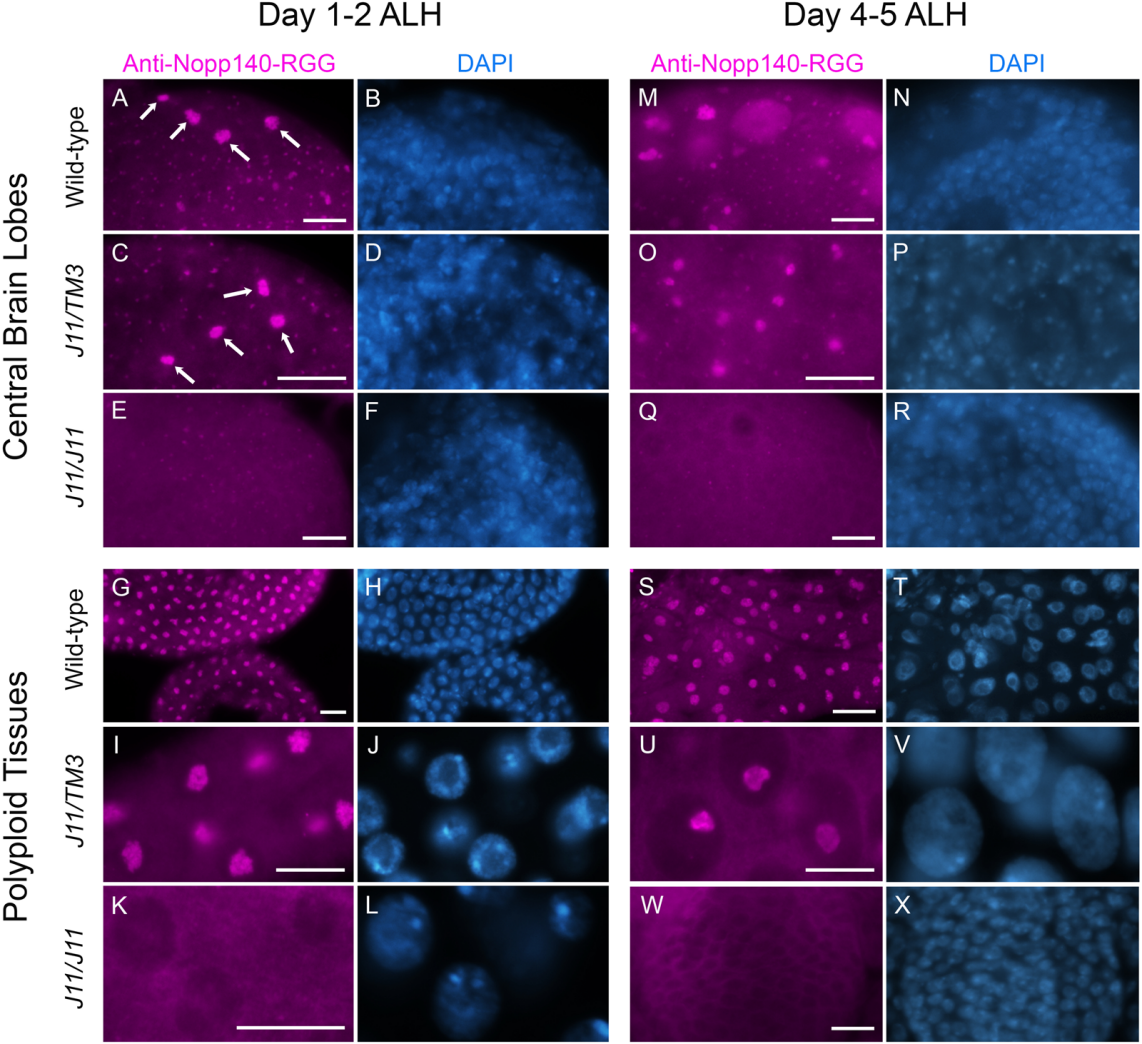
**Maternal Nopp140 protein is reduced in early *J11 Nopp140−/−* larval brains.** Central brain lobes and mid-gut tissues from *w^1118^* (wild-type) control, heterozygous *J11^non-DsRed^*/*TM3* and homozygous *J11^non-DsRed^* larvae at day 1–2 ALH (central brain lobes A–F, polyploid gut tissues G–L) and day 4–5 ALH (central brain lobes M–R, polyploid gut tissues S–X) were immuno-stained with anti-Nopp140-RGG. Arrows in panels A and C indicate four NBs per wild-type brain lobe with large nucleoli labeled with anti-Nopp140-RGG. Nuclei were counter-stained with DAPI. *n*=19 (*w^1118^*); *n*=13 (heterozygous *J11^non-DsRed^*/*TM3* larvae); *n*=23 (homozygous *J11^non-DsRed^*); two technical replicates. Scale bars: 10 µm.

Since the RT-PCR analyses showed that maternal *Nopp140* transcripts persisted in the homozygous *J11^DsRed^* larvae at day 1–2 ALH, we wanted to test if the Nopp140 protein could be detected in their brain and gut tissues as well. To do this, we immuno-stained homozygous *A7-J11^non-DsRed^* larvae and wild-type larvae with an antibody directed against Nopp140-RGG, one of the two Nopp140 isoforms in *Drosophila*. This antibody was raised against a synthetic peptide, the sequence of which is unique to the carboxyl tail region of Nopp140-RGG (see Cui and DiMario, 2007). At day 1–2 ALH, the anti-Nopp140-RGG antibody labeled nucleoli in homozygous *J11^non-DsRed^* larval brain and midgut (Fig. 3E,K), but at much lower levels compared to the same tissues in wild-type and *J11/TM3* larvae (Fig. 3, panels A and C for brain cells, G and I for midgut cells). Four large nucleoli per brain lobe were routinely detected in the posterior of wild-type and *J11/TM3* larval brain lobes 1–2 day ALH, and we speculated these were the MB NBs that do not undergo quiescence as do other NBs, but continue to divide throughout the embryo-to-larva transition (arrows in Fig. 3A,C). We did not observe this preferential labeling in homozygous *A7-J11^non-DsRed^* brains (Fig. 3E). By day 4–5 ALH, nucleolar labeling by anti-Nopp140-RGG was substantially reduced in homozygous *A7-J11^non-DsRed^* larval brain and midgut cells as compared to the wild-type and *J11/TM3* heterozygous tissues (Fig. 3, compare panel Q with panels M and O for brain cells, and panel W with panels S and U for midgut cells). The results indicated that at least the Nopp140-RGG isoform encoded presumably by maternal transcripts persisted in the first 2 days of homozygous *A7-J11^non-DsRed^* larval development, but then diminished in most cells as these *Nopp140−/−* larvae aged.

### Ultrastructural analysis

Central brain lobes from wild-type (4–5 day ALH) and *J11*/*J11* (6–7 day ALH) larvae were examined by TEM (Fig. S2, panels A and B). Neuronal cells in the *J11/J11* larvae contained ribosomes, but their density appeared reduced when compared to ribosome densities in wild-type neuronal cells. Ribonucleoprotein content within mitochondria of both wild-type and *J11/J11* neuronal cells appeared comparable by lead citrate staining, indicating that the overall lighter appearance of the cytoplasm in *J11/J11* cells was likely due to reduced ribosome content. Examination of midgut cells in these same *J11/J11* larvae (Fig. S2D) showed a significant decline in cytoplasmic ribosomes as compared to wild-type midgut cells (Fig. S2C). In particular, there is a tremendous loss of rough endoplasmic reticulum (rER, long arrows) in the *J11/J11* midgut cells. The appearance of unusual electron dense granules in the cytoplasm of *J11/J11* cells was consistent with our earlier report (He et al., 2015) describing these granules upon deletion of the *Nopp140* gene. Preliminary immuno-fluorescence evidence suggests these granules are related to processing (P) bodies (P. J. DiMario, unpublished observations/data).

### Embryonic and larval survivability with complete or partial elimination of Nopp140

**Fig. 4.**
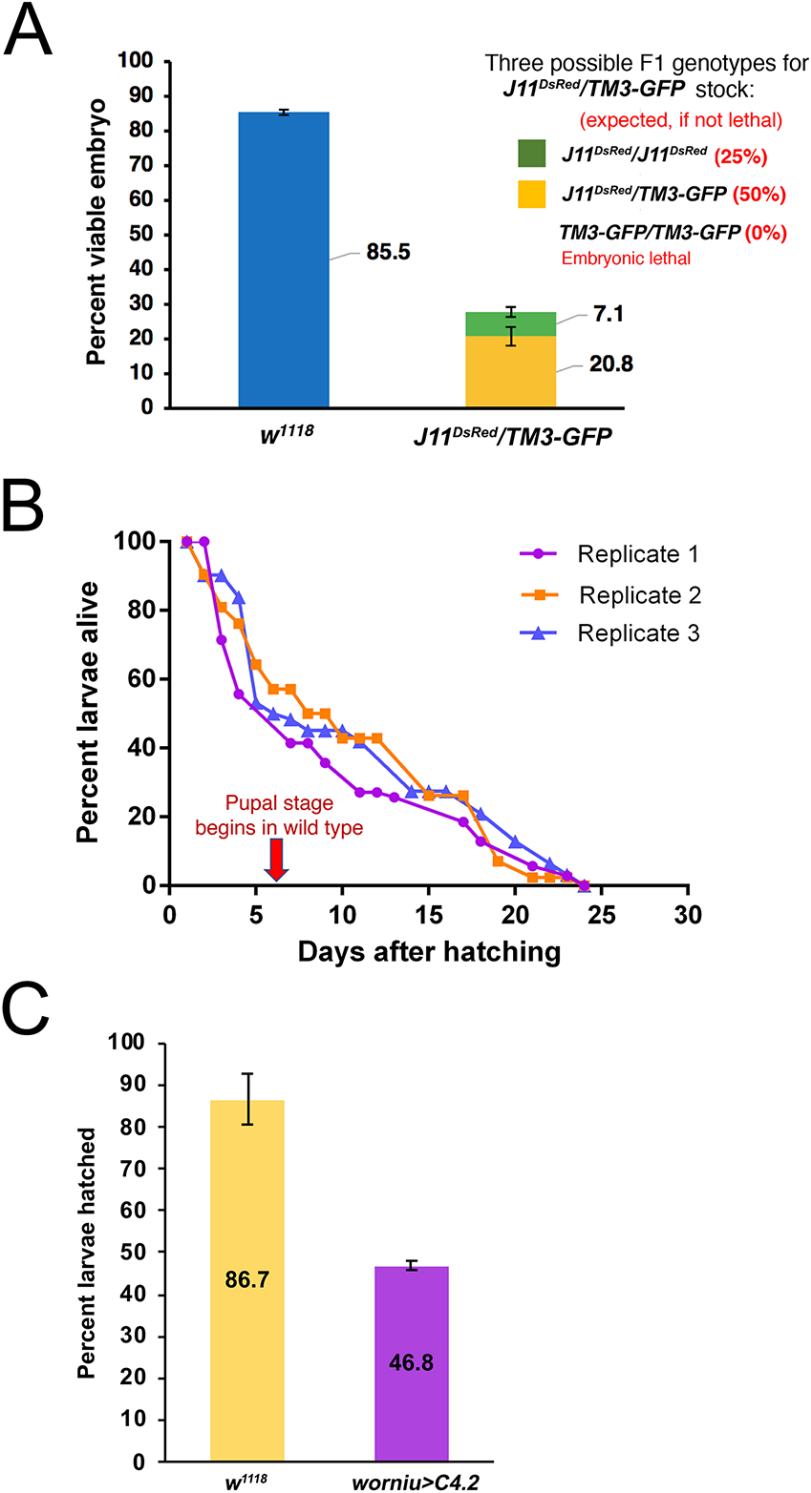
**Embryonic and larval survival upon complete or partial loss of Nopp140.** (A) Survival assays were performed for homozygous *J11^DsRed^* or heterozygous *J11^DsRed^/TM3-GFP* embryos, and for *w^1118^* control embryos. Freshly laid eggs were collected from well-yeasted juice plates (*n*=2; total number of embryos per replicate for *w^1118^*: 200 and 299, and for *J11^DsRed^/TM3-GFP* stock: 230 and 111). The number of hatched larvae were logged for the next 2 days, and the percent viable embryos was determined. Data shows the number of larvae hatched divided by the total number of embryos collected X 100%. (B) Plot shows three replicates (number of larvae per replicate: 70, 42, 62) of survival assay for homozygous *J11^DsRed^* larvae. Newly hatched larvae were collected from a well-yeasted juice plates, and the number of living larvae were recorded in the following days until all larvae had perished. (C) Embryonic lethality and larval survivability upon Nopp140 depletion by RNAi expression using the *worniu::GAL4* driver (specific for all embryonic and larval NBs) and *UAS::TComC4.2* (Nopp140-RNAi line; Cui and DiMario, 2007). Compared to 86.7% of the *w^1118^* embryos, only 46.8% of the collected embryos with Nopp140 depletion (*worniu-GAL4>C4.2*) hatched and developed into third instar larvae, after which all larvae developed into adults (not shown). *n*=3; total number of embryos collected per replicate for each genotype: 200, 265 and 330; Student's *t*-test: two-tailed with unequal variance, *P*-value=0.0069.

The *Nopp140* disruption lines were maintained using the third chromosome balancer, *TM3*, which carries a wild-type copy of the *Nopp140* gene. Embryos homozygous for *TM3* are non-viable, hence *inter se* crosses within the *J11^DsRed^/TM3* fly stock should produce 50% *Nopp140^DsRed^/TM3* larvae and 25% homozygous *J11^DsRed^* larvae (the number of hatched larvae/total number of eggs collected). However, if the disrupted *Nopp140* gene causes embryonic lethality, we would expect frequencies less than 50% and 25%, respectively. We found that only 20.8% of total eggs developed into larvae that were *J11^DsRed^/TM3* versus the expected 50% (Fig. 4A), and only 7.1% of the total eggs developed into larvae that were homozygous for *J11^DsRed^* versus the expected 25% (Fig. 4A). These data indicated that loss of *Nopp140* leads to partial embryonic lethality not only for the *homozygous J11^DsRed^* genotype as might be expected, but more interestingly for the heterozygous *J11^DsRed^/TM3* genotype. This observation indicated for the first time that the *Nopp140−/+* genotype exhibits haplo-insufficiency in *Drosophila*, reminiscent of the *Tcof1−*/+ genotype in the human TCS.

We earlier described growth arrest and lethality in second instar larvae that were homozygous for our original *pBac*-mediated *Nopp140^KO121^* deletion (He et al., 2015). Because of the particular *pBac* elements available at the time, we had to delete the 3′ end of the downstream gene, *P5CDh1* (He and DiMario, 2011), and this constantly forced us to control for the carboxyl truncation in P5CDh1, a mitochondrial matrix enzyme, when assessing the loss of Nopp140. Here, we assessed survivability of larvae homozygous for *J11^DsRed^* relative to wild-type larvae. Similar to our earlier findings (He et al., 2015), we found that ∼50% of the hatched homozygous *J11^DsRed^* larvae died by day 6 (which is when the pupal stage normally begins) (Fig. 4B). The remaining 50% remained as second instar larvae; they failed to grow or molt. The number of surviving homozygous *J11^DsRed^* larvae dwindled over time, but interestingly, some lingered up to day 24 (Fig. 4B).

While *J11/TM3* embryos displayed partial lethality, hatched *J11/TM3* larvae showed no readily apparent defects in growth or time of molting relative to wild-type larvae. In support of this observation, we depleted Nopp140 using the UAS-GAL4 system to express siRNAs. In the past, we showed that *daughterless::GAL4*>*UAS::TComC4.2* depleted ∼70% of the *Nopp140* transcripts (Cui and DiMario, 2007). Using the neuroblast-specific *worniu::GAL4* driver (*worniu-GAL4*>*UAS::TComC4.2*), we found embryonic survivability was ∼46%, while the wild-type embryo survival rate was ∼86% (Fig. 4C). Similar to the surviving *J11/TM3* larvae, surviving *worniu::GAL4*>*UAS::TComC4.2* larvae developed into viable and fertile adults. While the *worniu* promoter is active in all embryonic and larval NBs, its peak expression is in 6–12 h embryos, perhaps explaining the survivability of nearly half the *worniu::GAL4*>*UAS::TComC4* embryos beyond this embryonic stage.

### Brain hypoplasia upon nucleolar stress

**Fig. 5.**
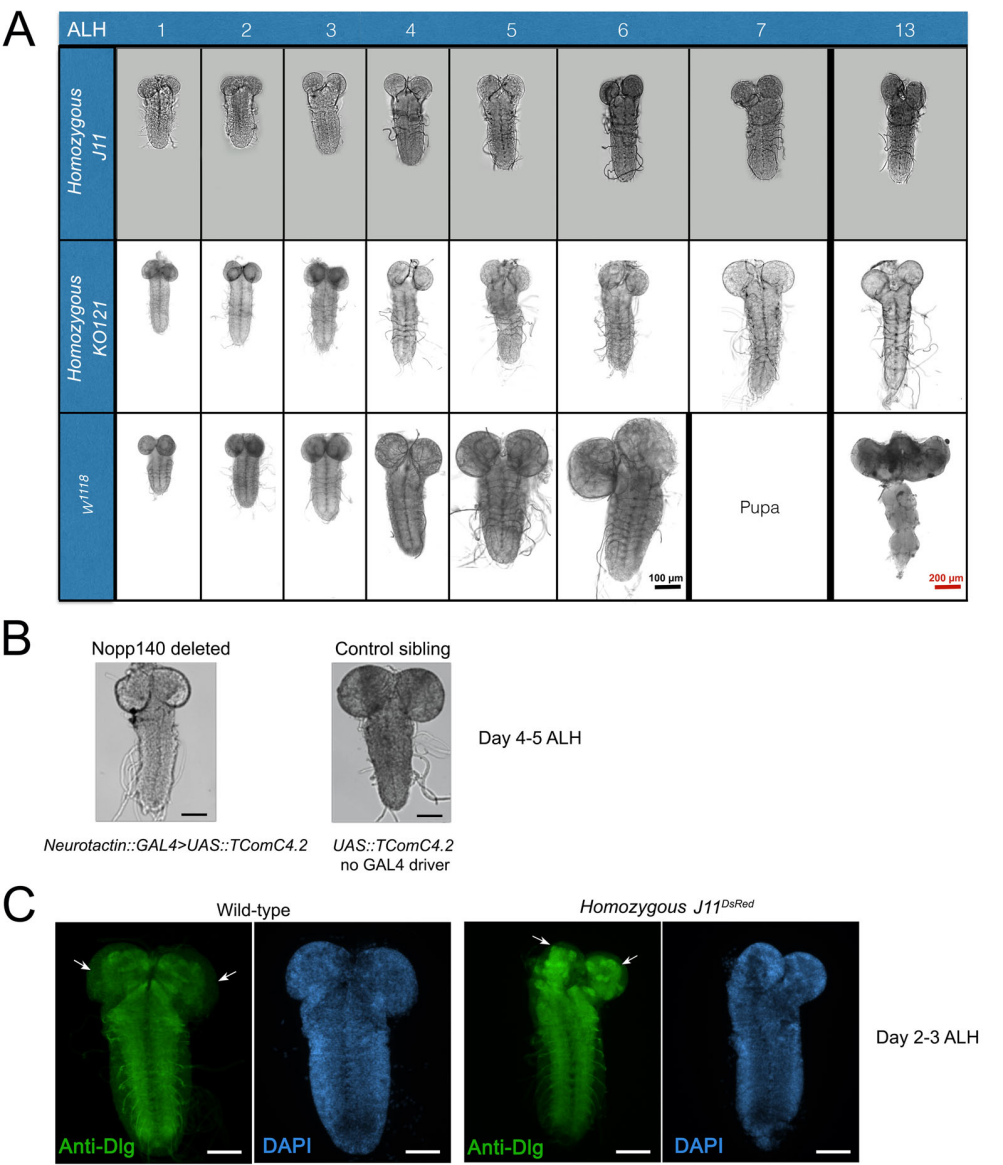
***Drosophila* larval brain development is impaired under nucleolar stress induced by the loss of Nopp140.** (A) Larval brain development in homozygous *KO121* (*Nopp140* gene deletion, He et al., 2015), homozygous *J11* (CRISPR-mediated *Nopp140* disruption), and wild-type (*w^1118^*) starting at day 1 after larval hatching (ALH) until day 7 and day 13 ALH. Homozygous *KO121* and *J11* larval brains at day 13 ALH are shown, but wild-type individuals have developed into adults by day 13 ALH, hence an adult fly brain is shown. (B) RNAi-depletion of Nopp140 using pan-neuronal GAL4 driver, *Neurotactin (Nrt)::GAL4*, and the *UAS::TComC4.2* (Nopp140 RNAi line) resulted in impaired larval brain development similar to that seen in Nopp140 homozygous deletion background. Representative larval brains from three independent crosses at day 4–5 ALH comparing Nopp140**-**depleted brains with control sibling brains (not expressing RNAi) are shown. Scale bars: 100 µm (C) Conventional fluorescence images of the neuropil immuno-stained with antibody against Discs large (Dlg; green) in second **i**nstar *w^1118^* control larvae and homozygous *J11^DsRed^* larvae at day 2–3 ALH. White arrows show unstained peripheral cell body layers, which are reduced in homozygous *J11^non-DsRed^* larvae. *n*=15 (wild-type); *n*=18 (homozygous *J11^non-DsRed^*); >3 technical replicates. Scale bars: 50 µm.

We found that larval brain development was severely impaired upon loss of Nopp140 either by gene disruption (i.e. homozygous *J11^DsRed^*) or by neuron-specific RNAi depletion. During the early larval stage (day 1–2 ALH), homozygous *J11^DsRed^* brains were morphologically comparable in size to brains from newly hatched wild-type larvae. The mutant's brain continued to grow from day 3–6 ALH, but more slowly compared to wild-type larval brains (Fig. 5A). Beyond day 5–6 ALH, however, homozygous *J11^DsRed^* larval brains failed to grow. This was similar to what we saw in our original *Nopp140^KO121^* deletion (He et al., 2015) (Fig. 5A). Likewise, brain growth was impaired in larvae upon RNAi-mediated depletion of Nopp140 using a pan-neuronal *GAL4* driver (*Neurotactin::GAL4>UAS::TComC4.2*) (Fig. 5B).

To determine where brain growth was impaired, we immuno-stained brains from homozygous *J11^DsRed^ larvae* and wild-type larvae at day 2–3 ALH with an antibody against *discs large* (anti-Dlg). This antibody labels axon bundles (the neuropil), but not the cell body mass which we counter-stained with DAPI. Neuropils within the two central brain lodes were reduced in homozygous *J11^DsRed^* brains as compared to wild-type brains, but there were no observable physical defects in the ventral nerve cord (VNC) neuropil of homozygous *J11^DsRed^* larvae when compared to wild-type larvae. Besides the reduced central brain lobe neuropils, we found the cell body mass of the central brain lobe was also reduced in homozygous *J11^DsRed^* brains when compared to wild-type brains (Fig. 5C).

### Reduced NB numbers and proliferation upon nucleolar stress

**Fig. 6.**
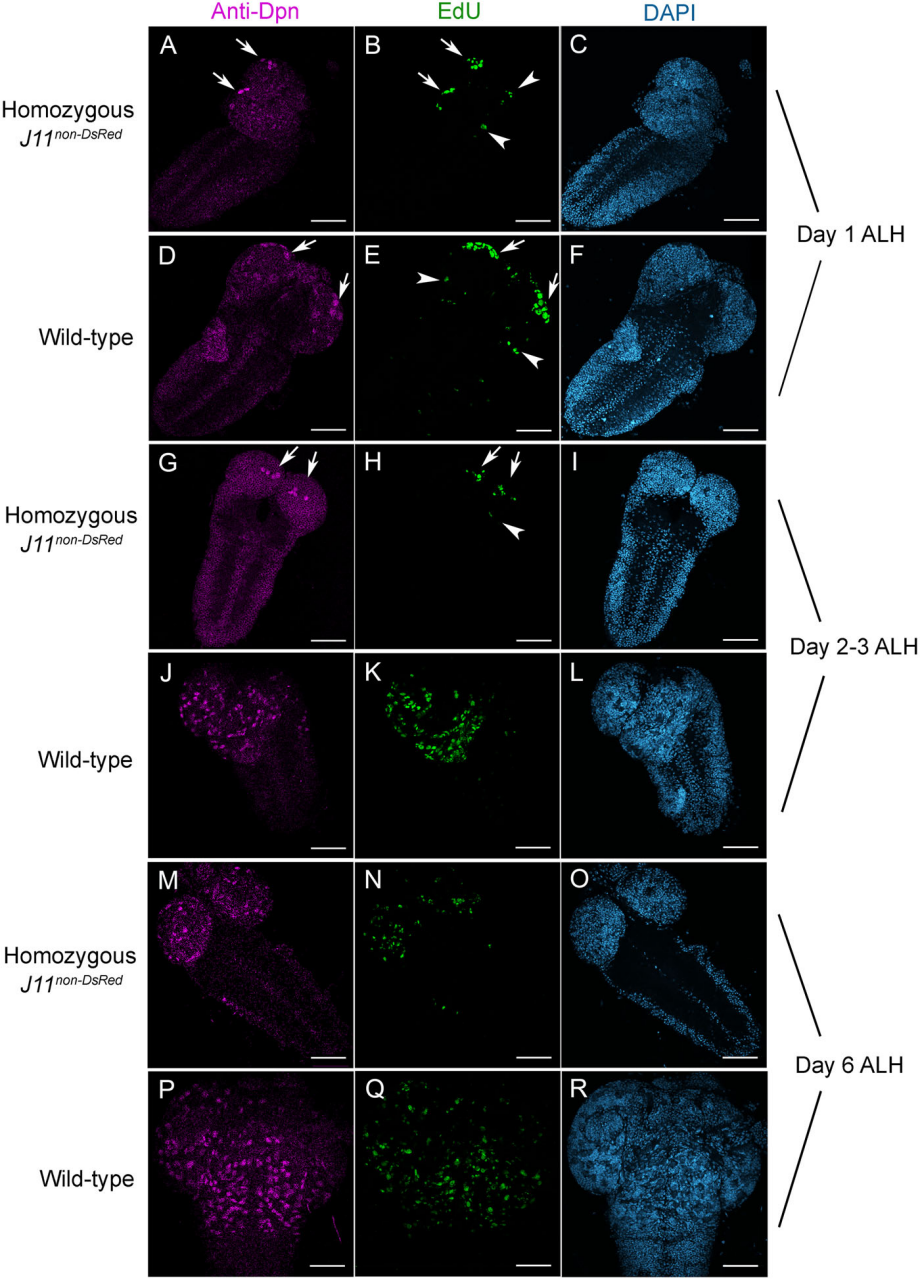
**Neuroblast proliferation is reduced upon nucleolar stress.** Confocal images of homozygous *J11^DsRed^* and control *w^1118^* larval brains at day 1 ALH (A–F), days 2–3 ALH (G–L), and day 6 ALH (M–R) are shown after EdU-labeling (Click-iT Alexa Fluor 488) followed by anti-deadpan (anti-Dpn) immuno-staining. Dpn-stained cells (magenta) are NBs. After a 30 min pulse, EdU-labeled S-phase cells (green) were committed to cell division. Arrows indicate four likely MB NBs that were EdU- and Dpn-positive and clustered near presumably the posterior end of the central brain (A,B,D,E,G,H). Arrowheads indicate a few EdU-positive cells, likely arising from AL MBs, at the lateral side of the central brain (B,E,H). *n*=10, 15 and 10 for days 1, 2–3, and 6, respectively, for both wild-type and homozygous *J11^DsRed^* samples; three technical replicates. Scale bars: 50 µm.

We hypothesized that the hypoplasia in *Nopp140−/−* larval brains was due to either a reduction in NB numbers, a reduction in the proliferative capacity of NBs, or both. To assess these possibilities, we first performed a Click-iT EdU labeling assay on living brains. EdU is a thymidine analog, which is incorporated into genomic DNA during S-phase of the cell cycle, and hence cells labeled by EdU are committed to cell division. We used 30 min EdU pulse-labeling assay in wild-type, homozygous *A7-J11^non-DsRed^* larval brains at 1, 2–3, and 6 days ALH (Fig. 6), and in heterozygous *A7-J11^non-DsRed^*/*TM3* larval brains at 1–2 and 4–5 days ALH (Fig. S5). After pulse-labeling, brains were fixed with paraformaldehyde and the incorporated EdU was fluorescently labeled by Click-iT chemistry. We then immuno-stained the brains with anti-deadpan to visualize the relative numbers and distribution of NBs within the brain lobes. Deadpan (Dpn) is a NB-specific transcription factor necessary for self-renewal.

Anti-Dpn labeling showed that NBs were present in homozygous *J11^non-DsRed^* larval brains from all age groups; however, their numbers were consistently reduced compared to either wild-type brains of similar age (Fig. 6; compare homozygous *J11^non-DsRed^* panels A, G, and M with wild-type panels D, J, and P), or to *J11^non-DsRed^/TM3* brains of similar age (Fig. S5). This suggested that the observed hypoplasia in the homozygous *J11^non-DsRed^* larval brains was due in part by fewer NBs.

Strikingly, homozygous *J11^non-DsRed^* larvae at day 1 and day 2–3 ALH consistently showed four NBs in each central brain lobe that were prominently labeled by anti-Dpn as compared to the surrounding Dpn-stained NBs (Fig. 6; arrows in panels A and G; Fig. S5, panel D). These four NBs were visible in wild-type and heterozygous *J11^non-DsRed^/TM3* brains as well, but only in brains from day 1–2 ALH larvae (Fig. 6D; Fig. S5D) as these four cells became less discernible in older larval brains among the many other NBs also present (Fig. S5G).

Subsets of EdU-positive cells in homozygous *J11^non-DsRed^*, wild-type and *J11^non-DsRed^ /TM3* brains were identified as NBs by the anti-Dpn nuclear staining. But EdU labeling also displayed the ganglion mother cells (GMCs) that were in S-phase. Overall, homozygous *J11^non-DsRed^* larval brains in all examined age groups showed fewer EdU-positive cells (both NBs and GMCs) as compared to wild-type brains (Fig. 6; homozygous *J11^non-DsRed^* panels B, H and N compared to wild-type panels E, K and Q) or to heterozygous *J11^non-DsRed^/TM3* brains (Fig. S5, homozygous *J11^non-DsRed^* panels E and K compared to *J11^non-DsRed^/TM3* panels B and H). This suggested a slower rate of NB proliferation in the homozygous *J11^non-DsRed^* larval brains. We did notice other EdU-positive cells in the lateral regions of the central brain lobes from both homozygous *J11^non-DsRed^* and wild-type larvae at day 1 ALH (Fig. 6, arrowheads in panels B and E). These should be the antennal lobe (AL) NBs. We occasionally detected them in central brain lobes of day 2–3 ALH homozygous *J11^non-DsRed^* (Fig. 6, arrowheads in panel H).

In larvae at day 2–3 ALH, we again observed only four NBs that co-labeled with both EdU and anti-Dpn in homozygous *J11^non-DsRed^* brains (Fig. 6, arrows in panels G and H). We predicted that these were the MB NBs based on their location and consistency in number. Wild-type larval brains, however, had far more EdU-positive and Dpn-positive cells, suggesting that the majority of wild-type NBs had now exited quiescence and started to proliferate as expected (Fig. 6, panels J and K).

Taken together, these observations indicated that upon nucleolar stress, a subset of NBs and GMCs, preferentially located within the MBs, proliferated in homozygous *J11^non-DsRed^* brains, but that these NBs gave rise to lineages that were comparatively smaller than those in wild-type brains under non-stressed conditions. Indeed, using an antibody against Prospero, a nuclear marker specific for GMCs and their descendent glia and neurons, we found significantly fewer GMC populations in the homozygous *J11^non-DsRed^* brains than in wild-type brains at day 1–2 and 6–7 ALH (Fig. S3). The apparent loss of most Type I and Type II neural lineages due to their inability to proliferate upon nucleolar stress likely contributed significantly to the brain hypoplasia. Additionally, we found reduced areas of cell nuclei in homozygous *J11^non-DsRed^* larval NBs and neurons compared to those in wild-type larval brains at day 2–3 ALH (Fig S4). This nuclear area reduction may further contribute to the brain hypoplasia in homozygous *J11^non-DsRed^* larvae.

### MB NBs are resilient to nucleolar stress

**Fig. 7.**
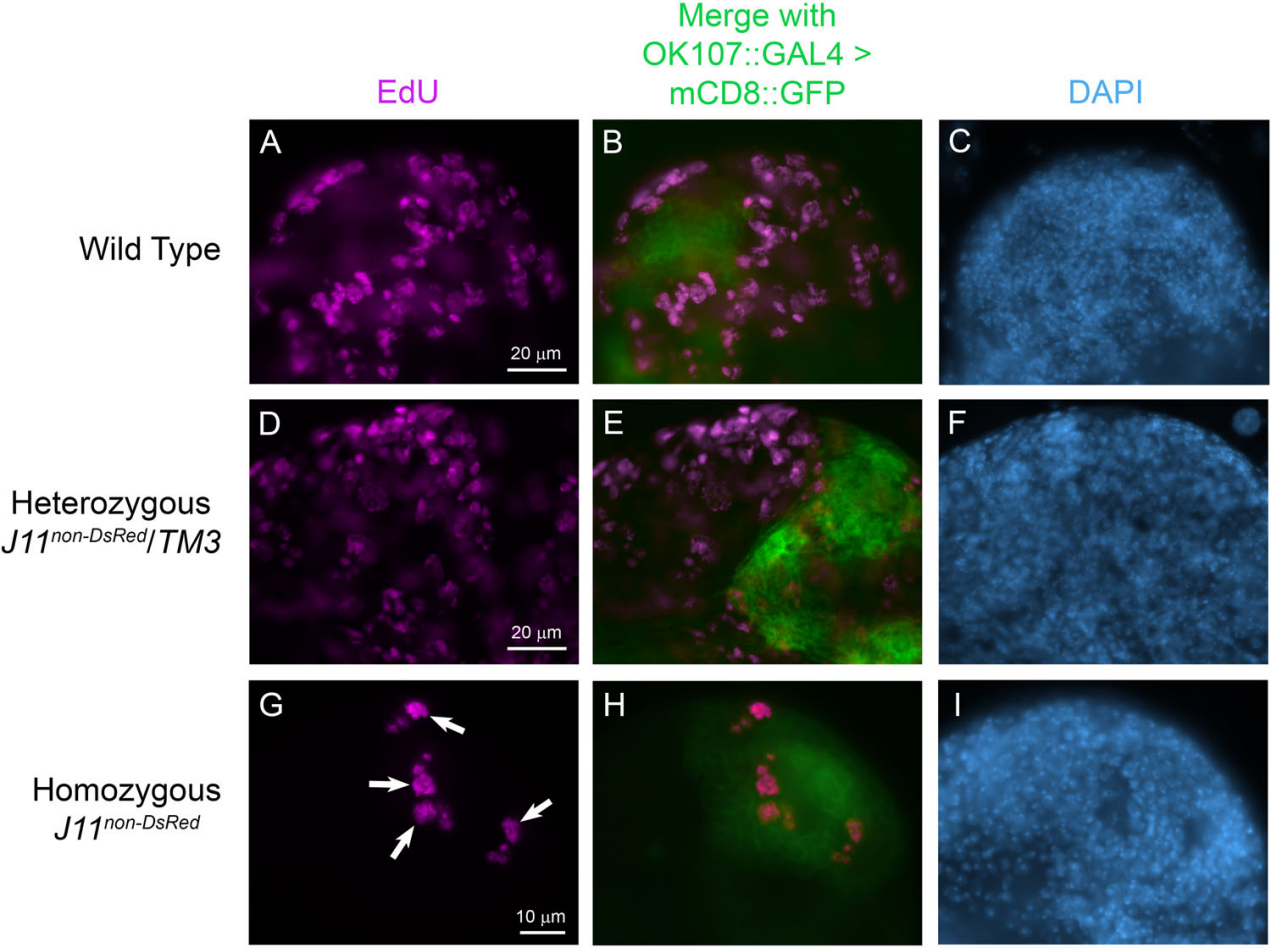
**MB NBs are resilient to nucleolar stress.** Larval brains from control *w^1118^* (wild-type), heterozygous *J11^non-DsRed^*/*TM3*, and homozygous *J11^non-DsRed^* larvae at day 3 ALH were used for 30 min EdU pulse labeling (Click-iT Alexa Fluor 594). Merged confocal images show EdU-labeled cells (magenta) nestled within the GFP-labeled MB lineage (green) near the central brain lobes. *n*=12 (control); *n*=10 (heterozygous *J11^non-DsRed^*/*TM3* larvae); *n*=20 (homozygous *J11^non-DsRed^*); three technical replicates. Scale bar dimensions are provided in panels A, D and G.

To test if the four EdU-positive NBs were in fact MB NBs, we used a MB lineage-specific *GAL4* driver to express a GFP-tagged plasma membrane reporter protein, mCD8-GFP (*OK107::GAL4*>*mCD8::GFP*), and again performed co-EdU labeling in brains from homozygous *J11^non-DsRed^* and control (wild-type and *J11^non-DsRed^*/*TM3*) larvae at day 3 ALH. EdU labeling again showed many S-phase cells in wild-type and *J11^non-DsRed^*/*TM3* larval brains; however, while a subset of these cells were typically found within the mCD8-GFP-positive MB-lineage cell cluster, it was often difficult to identify the MB NBs and descendent glia (Fig. 7, panels B and E). In homozygous *J11^non-DsRed^* larval brains at day 3 ALH, EdU-positive cells were always located within the MB lineage-cell cluster as identified by mCD8-GFP (Fig. 7, panel H). This suggested that the four Dpn-positive and EdU-positive NBs that we observed in homozygous *J11^non-DsRed^* larval brains at day 2–3 ALH (Fig. 6, panels G and H) were indeed MB NBs. The combined results of Figs 6 and 7 suggest that the MB NBs are more resilient to nucleolar stress induced by the loss of Nopp140 as compared to most other NBs within these brains. We base this MB NB resiliency on continued Deadpan labeling and co-EdU incorporation.

### MB NBs in *Nopp140−/−* larval brains retain fibrillarin in their nucleoli

**Fig. 8.**
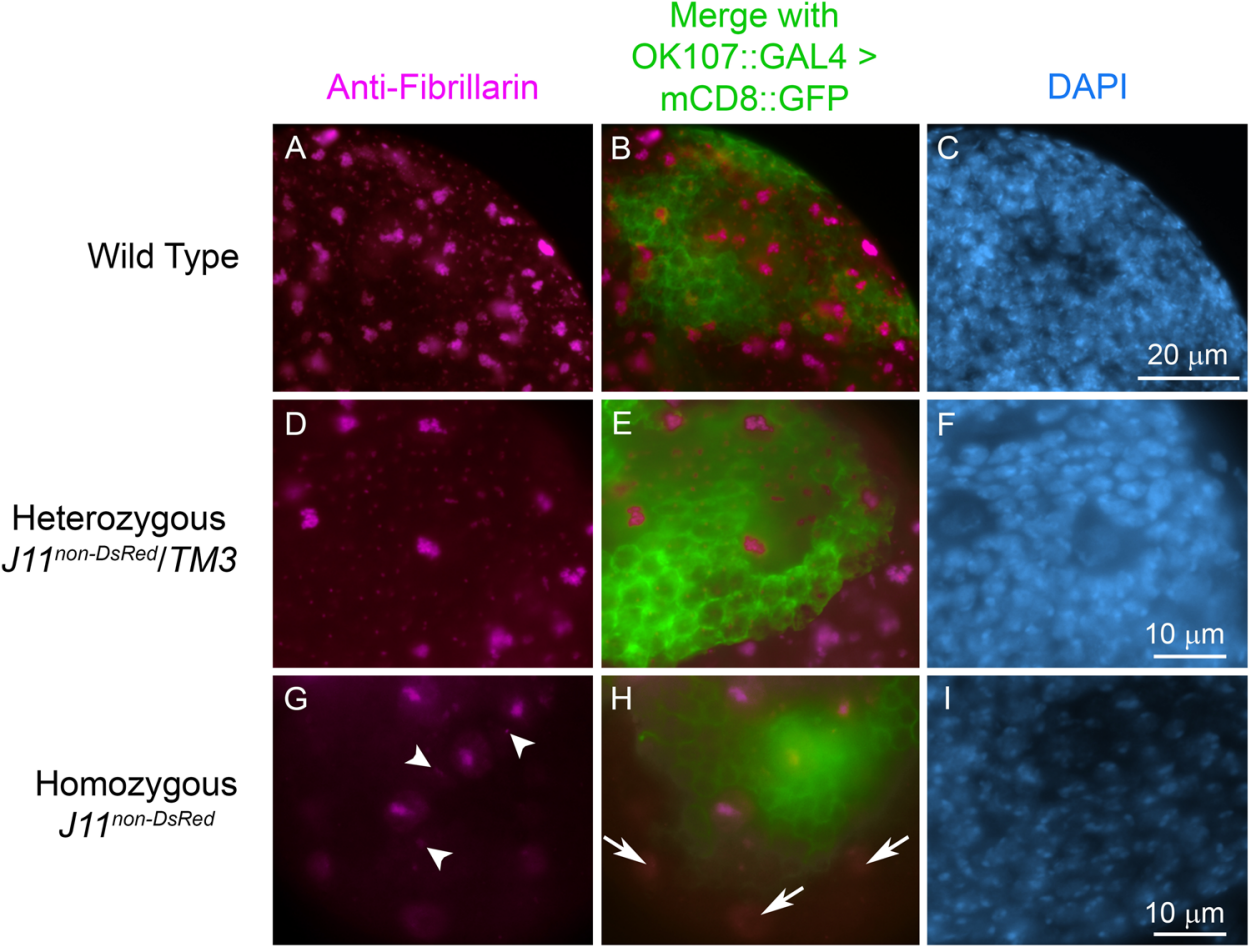
**MB lineage cells retain nucleolar fibrillarin under nucleolar stress.** Larval brains from control *w^1118^* (wild-type), heterozygous *J11^non-DsRed^/TM3*, and homozygous *J11^non-DsRed^* larvae at day 3 ALH were immuno-stained with anti-fibrillarin (magenta). Image panels B, E and H show anti-fibrillarin stained central brain lobes along with mCD8::GFP (green) expressed under MB-lineage specific *OK107::GAL4* driver in the same tissues. Four large nucleoli, presumably in the MB NBs, are apparent in the homozygous *J11^non-DsRed^* larval brains (G). Arrowheads in G show small nucleoli that reside in putative GMCs. Arrows in H indicate redistributed nucleolar fibrillarin in NBs residing outside of mCD8::GFP expressing MB lineage cells (green). *n*=10 (wild-type); *n*=14 (heterozygous *J11^non-DsRed^*/*TM3* larvae); *n*=10 (homozygous *J11^non-DsRed^*); two technical replicates. Scale bar dimensions are provided in panels C, F and I.

Nopp140 is a chaperone for C/D-box snoRNPs that catalyze 2′-*O*-methylation of pre-rRNA during ribosome biogenesis. Previous work in our lab showed that the C/D-box snoRNP methyl-transferase, fibrillarin, redistributes to the nucleoplasm upon complete loss of Nopp140 in larval tissues homozygous for the *Nopp140^KO121^* gene deletion. Loss of fibrillarin from the nucleoli caused a corresponding reduction in 2′-*O*-methylation of pre-rRNA, clearly indicating nucleolar dysfunction, even though gross nucleolar morphology and rDNA transcription remained normal (He et al., 2015). Since MB NBs, but not others, continue to divide in *Nopp140−/−* larval brains at day 2–3 ALH, we predicted that MB NBs might retain fibrillarin within their nucleoli, while other NBs and their lineages redistribute fibrillarin to the nucleoplasm. To test this, we used anti-fibrillarin to immuno-stain brains from homozygous *J11^non-DsRed^* and control (wild-type and *J11^non-DsRed^*/*TM3)* larvae at day 3 ALH. All three genotypes expressed mCD8::GFP in the MB (*OK107::GAL4>mCD8::GFP*). Anti-fibrillarin stained large nucleoli in neuroblasts and smaller nucleoli in neurons in the wild-type and *J11^non-DsRed^*/*TM3* larval brains (Fig. 8, panels A and D) with minimal nucleoplasmic labeling. Conversely, in homozygous *J11^non-DsRed^* larval brains, anti-fibrillarin failed to label the nucleoli in the majority of brain NBs outside the MBs. Instead, it labeled the nucleoplasm in the majority of these NBs. The exception was a small number of NBs, again just four and likely the MB NBs, usually located within the MB-lineage as marked by mCD8-GFP labeling (Fig. 8, panels G and H). These few cells showed clear nucleolar labeling with anti-fibrillarin, even though there was still some nucleoplasmic labeling (Fig. 8, panels G and H). Closer examination showed other smaller nucleoli in the near vicinity of the MB NBs (arrowheads in Fig. 8G). These may be nucleoli in the GMCs, but the identity of these cells awaits further analysis. Overall, this result indicates that the MB-lineage cells, and not others, are able to retain at least some nucleolar fibrillarin, indicating that their nucleoli are partially functional.

### A transcriptomics perspective

With nucleoli in MB NBs retaining fibrillarin, we used the NB-lineage specific transcriptome data set from Yang et al. (2016) to ask if MB NBs express more Nopp140 and fibrillarin relative to other NBs. We found the expression levels of four RBFs (Nopp140, fibrillarin, Nop56 and Nop60B) were generally higher in the MB NBs compared to the AL NBs and Type II NBs (Fig. S6A). As controls, we checked the data set for expression levels of *Deadpan*, which encodes a NB-specific transcription factor, *Prospero*, which encodes a NB- and GMC-specific transcription factor, and *Elav*, which encodes a *Drosophila* neuron-specific protein. As expected, *Deadpan* and *Prospero* expression levels were higher in NBs compared to neurons, and *Elav* expression was higher in neurons compared to NBs (Fig. S6B). Therefore, the transcriptomics data supports our experimental data, indicating further that RBFs are preferentially enriched in MB NBs.

## DISCUSSION

### Ontogenesis of the MB NB lineages

A wealth of knowledge exists for *Drosophila* neurogenesis making it possible to analyze developing brains at the level of individual NB lineages (Birkholz et al., 2015; Egger et al., 2008; Hartenstein and Wodarz, 2013; Homem and Knoblich, 2012; Urbach and Technau, 2003). This is particularly true for the MB. Insect MBs are central hubs for olfactory sensory input, learning, and memory (Thum and Gerber, 2019). Formation of the MBs begins during embryogenesis during which each MB NB differentially expresses unique combinations of the regulatory genes (Kunz et al., 2012; Yang et al., 2016). As far as we know, none of these gene products have direct links to ribosome production. During the embryo-to-larva transition, only the four MB NBs and one AL NBs continue to proliferate independently of dietary nutrients and PI3-kinase activity (Kunz et al., 2012; Prokop and Technau, 1994; Ito and Hotta, 1992; Lin et al., 2013; Sipe and Siegrist, 2017). The majority of other NBs, however, enter a period of quiescence (Kunz et al., 2012; Prokop and Technau, 1994; Ito and Hotta, 1992) and require dietary nutrients and PI3-kinase activity to exit quiescence ∼24 h after hatching (Lovick and Hartenstein, 2015; Sipe and Siegrist, 2017). During subsequent larval development, each MB NBs generates an almost identical repertoire of intrinsic Kenyon cells and continues to proliferate on into the pupal stages (∼85–90 h after pupa formation) (Ito and Hotta, 1992; Ito et al., 1997). As a result, the adult MB neuropil in each CB lobe is densely packed with around 2000–3000 Kenyon cells per lobe (Technau and Heisenberg, 1982; Aso et al., 2009).

### Identifying MB NBs as resilient to nucleolar stress

We asked if Type I and II neuroblasts, MB NBs, OL NBs, and AL NBs are affected variably upon nucleolar stress, as are stem cells and precursor cells in the human ribosomopathies. To induce nucleolar stress, we used CRISPR-Cas9 to disrupt the *Nopp140* gene, which encodes two isoforms that function as RBFs early in the nucleolar subunit assembly. While we could detect maternal *Nopp140* transcripts in 1–2 day *Nopp140*−/− larvae by RT-PCR, we failed to detect zygotically expressed *Nopp140* transcripts in these larvae (Fig. 2C). A polyclonal antibody directed against a unique peptide sequence within the RGG carboxyl domain of Nopp140-RGG showed reduced levels of this isoform in brain tissue of 1–2 day *J11/J11* larvae, and eventually no detectable fluorescence signal in brain tissue in 4–5 day *J11/J11* larvae (Fig. 3E and Q). An antibody directed against the carboxyl terminus of the other isoform, Nopp140-True, has proven much weaker in immuno-fluorescence assays, and was not used here. Neither antibody works well on western blots. *Drosophila* larvae homozygous for *J11^non-DsRed^* arrested in the second instar stage and showed smaller brains by day 4 ALH (Fig. 5A). While some of these homozygous larvae lingered to day 24 ALH, none of them survived (Fig. 4B). Compared to wild-type brains, homozygous *J11^non-DsRed^* larval brains at day 2–3 ALH had far fewer proliferating NBs (Fig. 6; Fig. S5). Data presented here indicate that the brain hypoplasia in homozygous *J11^non-DsRed^* larvae is due to both the loss of Type I and Type II NB divisions and to reduced sizes of the remaining NBs and neuronal cells (Fig. S4). Clonal analyses currently underway should provide detailed information on cell proliferation and sizes within the Nopp140-deficient clones versus surrounding, phenotypically normal cells.

On the other hand, anti-deadpan and EdU labeling of homozygous *J11^non-DsRed^* larvae showed that MB NBs in particular, and in some cases the AL NBs, proliferated through the embryo-to-larva transition, and they continued to proliferate at day 2–3 ALH and at day 4–5 ALH as other NB lineages remained arrested (Fig. 6). From this observation we conclude that MB NBs predominantly exhibited resilience to nucleolar stress caused by the loss of zygotic *Nopp140* gene expression. Thus, the MB NBs (and AL NBs) are inherently different in their proliferative schedules compared to the rest of the NBs in the *Drosophila* larval brain. This may explain in part their resilience to nucleolar stress; that is, continued NB proliferation and already high synthetic rates (e.g. ribosome production) may temporarily sustain the MB NBs upon nucleolar stress, while the other NBs may not be able to rekindle high synthetic levels as they exit quiescence (Bertoli et al., 2013).

### Phenotypes

For the first time, we showed that the *Nopp140* gene in *Drosophila* is haplo-insufficient where *J11^DsRed^/TM3* displayed embryonic lethality (Fig. 4A). This was a surprise since a previous segmental aneuploidy study indicated no haplo-insufficiency genes existed in cytological region 78F4 of the left arm of chromosome 3 (Lindsley et al., 1972). Haplo-insufficiency of the *Drosophila Nopp140* gene would be analogous to haplo-insufficiency of the human *Tcof1* gene, which encodes treacle, a vertebrate RBF related to Nopp140 in peptide structure and function in early subunit assembly. Loss of treacle in *Tcof1+/−* human embryos results in TCS, a ribosomopathy leading to apoptosis in select embryonic neural crest cells ultimately leading to the craniofacial birth defects.

Earlier work in our lab showed that complete loss of Nopp140 in *Drosophila* induced nucleolar stress with the redistribution of the C/D box methyl-transferase, fibrillarin, to the nucleoplasm (He et al., 2015). Here, we showed that *Nopp140* transcripts and at least the Nopp140-RGG isoform were reduced, but not completely absent in early larvae homozygous for the disrupted *Nopp140* allele, *J11^non-DsRed^* (Figs 2 and 3). Interestingly, each wild-type central brain lobe showed four anterior cells that appeared to contain more Nopp140-RGG than other cells within the same lobes (Fig. 3). The observation suggested that MB NBs contain more Nopp140 than do other NBs. We then showed that mCD8::GFP-labeled MB lineages in homozygous *J11^non-DsRed^* larvae retained nucleolar fibrillarin, whereas fibrillarin was noticeably redistributed to the nucleoplasm in the majority of other NBs (Fig. 8). This latter observation indicated that nucleoli in the MB lineages preferentially retained more RBFs and perhaps maintained functional production of ribosomes longer, although to what extent requires future molecular analyses.

### Differential RBF expressions

Most cells within the central brain lobes of homozygous *J11^non-DsRed^* larvae 1–2 day ALH showed reduced anti-Nopp140-RGG labeling compared to brain cells in similarly aged wild-type larvae. Interestingly, wild-type larvae clearly showed four cells, identified as MB NBs, per central brain lobe with prominent anti-Nopp140-RGG labeling (Fig. 3). The observation suggests that wild-type MB NBs contain more zygotically expressed Nopp140 than do other NBs. Recent findings supporting this notion show that various RBFs such as treacle, fibrillarin, Nop56, mbm and NS3 are overexpressed in stem cell and progenitor cell populations (Brombin et al., 2015; Watanabe-Susaki et al., 2014; Wang et al., 2013; Johnson et al., 2018; Hovhanyan et al., 2014; Hartl et al., 2013; Dixon et al., 2006), and that the quantity and spatiotemporal expression of RBFs can vary in different stem cell or progenitor cell populations (Weiner et al., 2012; Bouffard et al., 2018).

Related to differential expression of RBFs in stem cells and progenitor cells is the perplexing problem of why some larvae homozygous for the disrupted *Nopp140* gene survive up to day 24 ALH (Fig. 4B). While we have yet to pursue this question rigorously, we suspect these *Nopp140−/−* individuals may inherent more maternal *Nopp140* mRNA and/or protein, and this may be a function of the nutritional state and health of the mothers. Earlier work in our lab (McCain et al., 2006) followed maternally expressed exogenous GFP-Nopp140 protein into embryogenesis, and noted that it lingered for several days. Individual *Nopp140−/−* embryos that inherited extra maternal *Nopp140* transcripts or protein would likely produce more ribosomes and survive longer.

### The possibility of heterogeneous ribosomes

Ribosomes are not all the same even within a single cell (Xue and Barna, 2012; Guo, 2018). Werner et al. (2015) showed that the translation program of human embryonic stem cells (hESCs) differentiating into neural crest cells changed after the depletion of KBTBD8, a substrate adapter for the vertebrate-specific ubiquitin ligase, CUL3. CUL3 mono-ubiquitylates human Nopp140 (NOLC1) and treacle, and forms a Nopp140-treacle platform that connects RNA Pol I machinery with ribosome modification factors. Based on these results, the authors hypothesized that the change in translational profile was the result of differential alteration of ribosomes. Modifications such as rRNA pseudouridylation and methylation, or phosphorylation and ubiquitylation of ribosomal proteins or ribosome-associated factors may ultimately contribute to translational control of gene expression (Sloan et al., 2017). Thus, the abundance of Nopp140 in different *Drosophila* NBs could potentially lead to differential modifications of the ribosome pools, and thus changes in the translational profile in different NBs.

Finally, transcriptome profiles (Fig. S6) of the different *Drosophila* larval brain NBs support our finding that RBFs Nopp140 and fibrillarin exist in higher levels in the MB NBs. The transcriptomics suggest that Nop56 and Nop60B may also exist in higher levels in MB NBs. Are there heterogeneous pools of ribosomes within a *Drosophila* larval brain? The *Drosophila* nervous system should serve well to explore differential threshold requirements for ribosome production and the diversity of resulting ribosome pools.

## MATERIALS AND METHODS

### Fly stocks

Fly lines used in this study included: *w^1118^* (used as a wild-type control, Bloomington stock #3605), the third chromosome balancer stock w*; Sb^1^/TM3, P{ActGFP}JMR2, Ser^1^ (referred to as *TM3-GFP*, Bloomington stock #4534), y^1^ M{nos-Cas9.P}ZH-2A w* (referred to as *nanos-Cas9*, Bloomington stock #54591 provided by Fillip Port and Simon Bullock, MRC Laboratory of Molecular Biology), w*; P{GawB}OK107 ey^OK107^/In(4) ci^D^, ci^D^ pan^ciD^ sv^spa-pol^ (referred to as *OK107-GAL4*, Bloomington stock #854), w*; *P{wor.GAL4.A}2; Dr^1^/TM3, P{Ubx-lacZ.w^+^}TM3, Sb^1^* (referred to as *worniu-GAL4*, Bloomington stock #56553), *w^1118^; P{GMR37H04-GAL4}attP2* [referred to as *Scabrous* (*Sca*)*-GAL4*, Bloomington stock #49969], *w^1118^; P{y[+t7.7] w[+mC]=GMR38F05-GAL4}attP2* [referred to as *Neurotactin (Nrt)-GAL4*, Bloomington stock #49383], y^1^ w*; P{w^+mC^=UAS-mCD8::GFP.L}LL5, P{UAS-mCD8::GFP.L}2 (referred to as *UAS-mCD8-GFP*, Bloomington stock #5137), *KO121 Nopp140* gene deletion line (He et al., 2015), and the *UAS-TComC4.2 Nopp140* RNAi line (Cui and DiMario, 2007). Flies were maintained in the laboratory at room temperature (22–24°C) on standard cornmeal-molasses medium. All applicable international, national, and/or institutional guidelines for the care and use of animals were followed.

### Homology directed insertion of *DsRed* into *Nopp140*

We used CRISPR-Cas9 and HDR to insert the *DsRed* gene into the second exon of the *Nopp140* gene. The CRISPR optimal target finder tool (http://targetfinder.flycrispr.neuro.brown.edu/) provided 271 gRNA target sites, each 20 nt in length excluding the NGG PAM sequence. Among these, six gRNAs had zero off-targets in coding regions of the *Drosophila* genome*.* The gRNAs were additionally verified to have no off-targets by the TagScan tool (Genome-wide Tag Scanner; https://ccg.epfl.ch//tagger/tagscan.html), and the Cas-OFFinder tool (Bae et al., 2014). Two gRNA targets, gRNA#52 (5′GGGCTTTGCCGGTTCTTCCTCGG on the minus strand of *Nopp140*; with the PAM sequence underlined) and gRNA #99 (5′CAAGTTGGCTCCTGCTAAGAAGG on the plus strand of *Nopp140*), were chosen and used for CRISPR gene editing. Successful CRISPR-Cas9 cleavage at both gRNA target sites would delete 321 bps from the second exon.

To express these gRNAs, sense and anti-sense oligos that included *BbsI* restriction site overhangs were prepared for both gRNAs by Integrated DNA Technologies (IDT; see Table 1 for gRNA sequences). Mixtures of sense and anti-sense oligos for each gRNA were annealed (heated at 95°C for 5 min, and then cooled to room temperature over 1 h in 1× ligation buffer). The resulting double-strand DNAs were ligated separately into *pCFD3-dU6:3gRNA* at the *BbsI* site. *pCFD3-dU6:3gRNA* was a gift from Simon Bullock [Addgene plasmid # 49410; http://n2t.net/addgene:49410; RRID:Addgene_49410; (Port et al., 2014; Ren et al., 2013)]. The resulting plasmids are referred to as *gRNA#55* and *gRNA#99* (Fig. 2A).

**Table 1.**
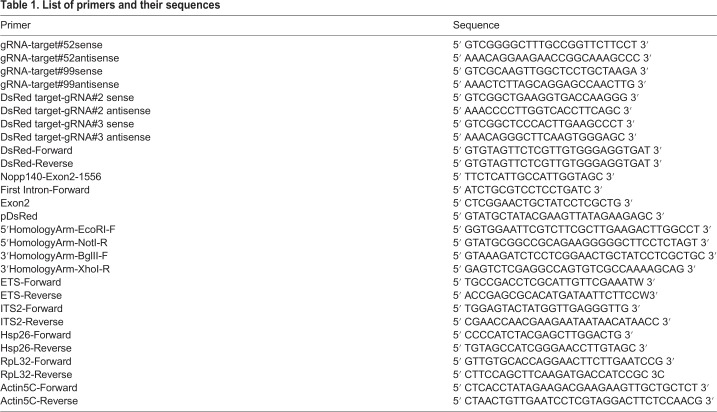
**List of primers and their sequences**

To mark the disrupted *Nopp140* gene, the *DsRed* gene was inserted at the Cas9-mediated deletion site by HDR. We used the donor plasmid, *pDsRed-attP*, which was a gift from Melissa Harrison, Kate O'Connor-Giles, and Jill Wildonger (University of Wisconsin-Madison) (Addgene plasmid # 51019; http://n2t.net/addgene:51019; RRID:Addgene_51019; Gratz et al., 2014). We followed general guidelines (Gratz et al., 2014) to insert the homology arms into the multiple cloning sites available on either side of the *DsRed* gene in *pDsRed-attP*. The 5′ and 3′ homology arms from the *Nopp140* second exon were prepared by PCR using forward and reverse primers listed in Table 1. These homology arms flank the 321 bp deletion region described in the preceding paragraph (Fig. 2A). We first inserted the 421 bp 3′ arm into *pDsRed-attP* at the *BglII* and *XhoI* sites upstream of the *DsRed* gene, and then inserted the 500 bp 5′ arm at *NotI* and *EcoRI* sites downstream of the *DsRed* gene. The orientation of the homology arms relative to *DsRed* should insert the *DsRed* sequence by HDR such that transcription of *DsRed* is in the opposite direction relative to transcription of the *Nopp140* gene. The final plasmid is referred to as *pDsRed*-*Dono*r (Fig. 2A).

### NHEJ disruption of *DsRed* gene inserted within *Nopp140* second exon

To mutate the *DsRed* gene within the *Nopp140* gene in the *J11 DsRed* fly line, we used Cas9 endonuclease expressed from the *pBS-Hsp70-Cas9* plasmid, a gift from Melissa Harrison, Kate O'Connor-Giles, and Jill Wildonger (Addgene plasmid # 46294; http://n2t.net/addgene:46294; RRID:Addgene_46294; Gratz et al., 2013). To find gRNA target sites within the *DsRed* gene, we again used the CRISPR optimal target finder tool, which yielded 38 gRNA target sites that were 18-nt in length. Twelve of the 38 gRNA targets had no matches to the *Drosophila* genome. Among the twelve gRNA targets, we chose gRNA#2 (5′GCTGAAGGTGACCAAGGGCGG on the plus strand of *DsRed*) and gRNA#3 (5′GCTCCCACTTGAAGCCCTCGG on the minus strand of *DsRed*). Sense and anti-sense oligos for each gRNA target site were prepared by IDT (see Table 1 for sequences). Each double stranded DNA encoding the respective gRNAs was separately ligated into the *pCFD3-dU6:3gRNA* plasmid at the *BbsI* restriction site following the same procedures described above for the preparation of *gRNA#52* and *gRNA#99* plasmids. The resulting plasmids for *DsRed* gene mutagenesis are *gRNA#2* and *gRNA#3*.

### *Drosophila* embryo injections

All plasmids used for embryo injections were extracted from transformed *Escherichia* *coli* cells using a plasmid Midiprep kit from Thermo Fisher Scientific. To disrupt the *Nopp140* gene, the plasmid injection mixture contained 15 ng µl^−1^ of *gRNA#52*, 15 ng µl^−1^ of *gRNA#99*, and 230 ng µl^−1^ of *pDsRed-Donor*. The mixture was injected into homozygous *nanos-Cas9* transgenic embryos. To disrupt the *DsRed* gene, the CRISPR injection mixture contained 75 ng µl^−1^ of *gRNA#2*, 75 ng µl^−1^ of *gRNA#3*, and 350 ng µl^−1^ of *pBS-Hsp70-Cas9*. This mixture was injected into *J11 DsRed/TM3-GFP* embryos. All injections were performed by GenetiVision Corporation (Houston, TX, USA).

### PCR verification of Homology Directed Cas9-mediated donor sequence insertion

Approximately 30 healthy well-fed adults were homogenized in 100 mM Tris-HCl (pH 7.5), 100 mM EDTA, 100 mM NaCl and 0.5% SDS, followed by 30 min incubation at 70°C. Genomic DNA was precipitated in a 1:2 ratio of 5 M KOAc:6 M LiCl on ice for 10 min, followed by phenol-chloroform purification and ethanol precipitation. PCR reactions contained 20–70 ng of genomic DNA, 0.40 µM of each primer, 0.20 mM of each dNTP, 0.50 mM of MgCl_2_, 1 X Phusion GC Buffer and 0.40 unit of Phusion high-fidelity DNA polymerase (M0530S, New England Biolabs). Amplification was performed in a Bio-Rad C1000 Thermal Cycler (cycling conditions: 32 cycles of denaturation for 30 s at 95°C, annealing for 30 s at 62°C, and elongation at 72°C for 1 min 20 s). Primers used for PCR verification were *DsRed-Reverse* and *Nopp140-Exon2-1556*. Their sequences are provided in Table 1.

### Sequence analyses

PCR products were extracted from agarose gels using phenol-chloroform, ethanol precipitated, and then sequenced using a BigDye Terminator Cycle Sequencing kit v.3.1 and an ABI 3130XL Genetic Analyzer (Applied Biosystems). Sequencing primers are indicated wherever the sequence reads are provided. Sequences were analyzed and aligned using CLC Sequence Viewer (Qiagen Bioinformatics).

### RT-PCR analysis

Larvae at day 1–2 after larval hatching (ALH) or day 5–7 ALH were collected from well-yeasted grape juice plates, placed into an Eppendorf tube, and rinsed with distilled water to remove yeast and other debris. Total RNA was extracted from wild-type or *Nopp140−/−* larvae using TRIzol (Invitrogen) according to the manufacturer's recommendations. First-strand cDNA synthesis was performed using M-MuLV Reverse Transcriptase (NEB M0253S) according to the manufacturer's recommendations with either oligo(dT) primers or gene-specific reverse primers (same as the reverse primers used in PCR). Oligo(dT) primers were used to synthesize the first-strand cDNA of *Hsp26*, *RpL32* and *Actin5C.* Gene-specific reverse primers were used for the *ETS and ITS2* regions of pre-ribosomal RNA. Specific forward and reverse PCR primers are described in Table 1.

### EdU labeling

For 5-ethynyl-2-deoxyuridine (EdU) labeling, larval brains were dissected in PBS (without detergent or azide), and within 5 min of dissection, the brains were incubated with 20 µM EdU in PBS for 30 min at room temperature. The tissues were then fixed for 30 min at room temperature in Buffer B (16.7 mM KH_2_PO_4_/K_2_HPO_4_, 75 mM KCl, 25 mM NaCl, 3.3 mM MgCl_2_, pH 7.0–7.2) with 2% paraformaldehyde (from a freshly prepared 10% stock) (de Cuevas and Spradling, 1998). EdU incorporated into S-phase cells was detected by a Click-iT Alexa Fluor 488 EdU imaging kit (Invitrogen) according to the manufacturer's recommendations. EdU was also detected by Alexa Fluor 594-Azide (Product No.1295, AF 594 Azide from Click Chemistry Tools) used with the reagents provided by Invitrogen Click-iT EdU imaging kit. Following EdU labeling, the larval brains were immuno-stained with antibodies followed by DAPI counterstaining.

### Immuno-staining and fluorescence microscopy

Larval brains and other tissues were dissected directly into fixation Buffer B described in the previous section. Tissues were fixed for 30–35 min total starting from the point when the dissection commenced. All washings were done with PBS with 0.1% TX-100 detergent. The blocking solution was 3% BSA prepared in PBS with 0.1% TX-100 which was also used for preparing dilutions of primary and secondary antibodies. In all cases, tissues were incubated in the primary antibody overnight at 4°C on a shaker, and in the secondary antibody for 4 h at 4°C on a shaker. Primary antibodies included the polyclonal guinea pig anti-Nopp140-RGG (Cui and DiMario, 2007) used at 1:100, a rat monoclonal anti-deadpan (Abcam, 195173, stock 1 mg ml^−1^) used at 1:250, the mouse monoclonal anti-fibrillarin mAb 72B9 (Reimer et al., 1987; hybridoma supernatant used without dilution), the mouse monoclonal anti-prospero (deposited at the DSHB by C.Q. Doe; DSHB Hybridoma Product: Prospero MR1A) used at 1:50, and the mouse monoclonal anti-discs large (dlg) (deposited at the DSHB by C. Goodman; DSHB Hybridoma Product: 4F3 anti-discs large) used at 1:30. Secondary antibodies included the Alexa Fluor 546 conjugated goat anti-rat (A-11081, Thermo Fisher Scientific) used at 1:1000, the Alexa Fluor 594 conjugated goat anti-guinea pig (A-11073, Thermo Fisher Scientific) used at 1:500, and the DyLight 488 conjugated goat anti-mouse (35503, Thermo Fisher Scientific) used at 1:500. Tissues were counter-stained with 4′,6-diamino-2-phenylindole (DAPI, Polysciences) at 1 µg ml^−1^. To image the tissues, we used either a conventional fluorescence microscope, a Zeiss Axioskop equipped with a SPOT RTSE digital camera, or a Leica SP8 Confocal Microscope equipped with the White Light Laser system in the Shared Instrumentation Facility (SIF) at Louisiana State University.

### TEM

Wild-type and *Nopp140−/−* larval tissues were prepared for transmission electron microscopy (TEM) using standard techniques as described by He et al. (2015). Images were captured using a JEOL 1400 TEM in the Shared Instrumentation Facility (SIF) at Louisiana State University.

### Determination of nuclear area

The 2D confocal images of the *Nopp140−/−* and wild-type larval brains at day 2–3 ALH were analyzed using Fiji software. After setting scale for each image, the free hand selection tool was used to draw outlines of each nucleus, and the nuclear area was subsequently recorded. Deadpan-stained larval brains were used to determine the nuclear area of NBs. Neuronal nuclear area was obtained from the DAPI-stained larval brains. The nuclear areas were plotted into a box-scatter plot using Microsoft Excel, and a Student's *t*-test (one-tailed) was performed on the data.

## Supplementary Material

Supplementary information
