# Supplementary material for: Nucleolar stress in Drosophila neuroblasts, a model for human ribosomopathies
Source: Biol Open. 2020 Apr 28;9(4):bio046565. doi: 10.1242/bio.046565 (PMC7197718; doi:10.1242/bio.046565)
Supplement: Supplementary information [file biolopen-9-046565-s1.pdf]

Supplementary Information

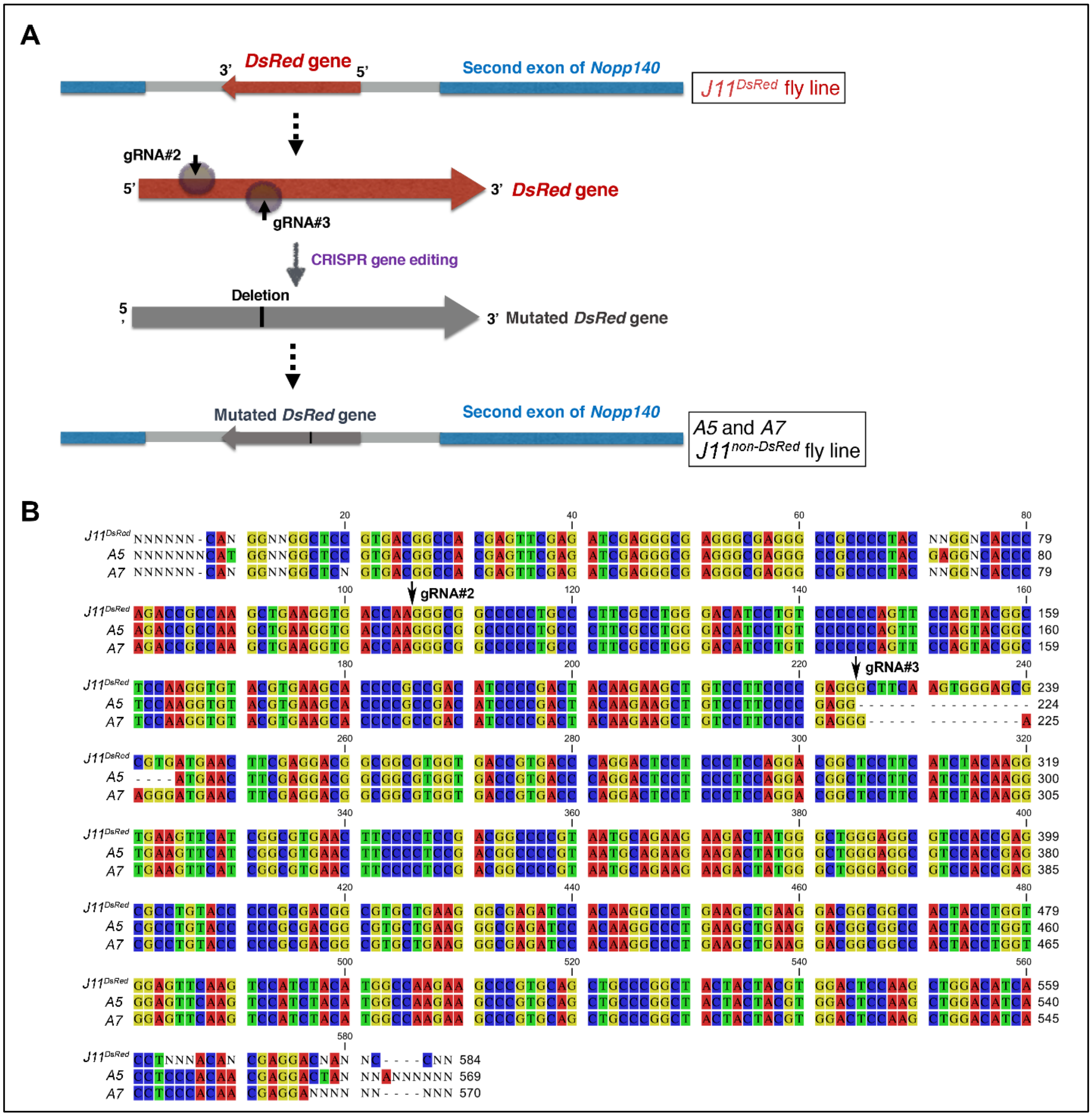

Fig. S1. **CRISPR-mediated *DsRed* gene disruption in the *J11*^{DsRed} fly line.** A) The *DsRed* gene disruption was achieved by targeting two sites within the gene with two gRNAs, gRNA#2 and gRNA#3. Small deletions were detected at the gRNA#3 target site in two of the *J11*^{DsRed} fly lines, *A5* and *A7*, that were isolated after the CRISPR gene editing event. B) The *DsRed* genomic region was sequenced in *J11*^{DsRed} fly lines *A5* and *A7* in both directions using DsRed-Forward and DsRed-Reverse primers. Sequence comparison revealed that the *A5* line had a 20 bp deletion at gRNA#3 target site, whereas the *A7* line had a 14 bp deletion at the same site as

Biology Open • Supplementary information

A5 line. The gRNA#2 and gRNA#3 target sites are indicated by arrows.

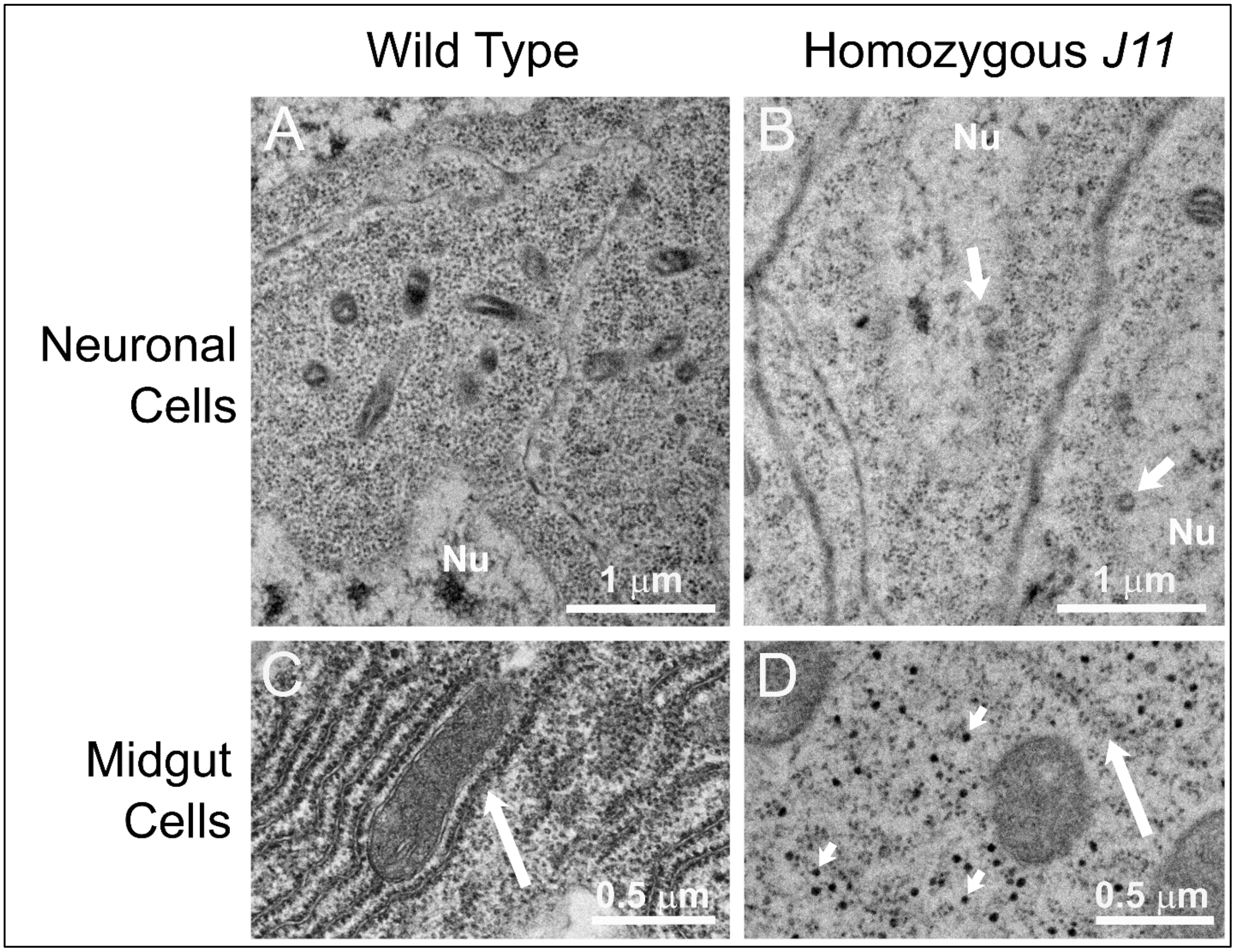

Fig. S2. **TEM analysis of wild type and homozygous *J11* larval brain and midgut cells. A** Neuronal brain cells from day 4-5 (third instar) wild type larvae contained ample ribosomes. Mitochondria in tangential or cross section were evident in the cytoplasm. Nuclei (Nu) contained patches of electron dense heterochromatin. **B** Most neuronal cells from day 6-7 homozygous *J11* larvae showed reduced ribosome levels compared to wild type neuronal cells. Arrows point to nuclear pore complexes. **C** Wild type midgut cells at all stages of larval development contained ample ribosomes. Arrow points to rER heavily populated with ribosomes. **D** Midgut cells from day 6-7 homozygous *J11* larvae were deficient in ribosomes. Long arrow indicates rER partially populated with ribosomes. Short arrows indicate unusual electron dense granules originally described by He et al. (2015) upon deletion of the *Drosophila Nopp140* gene. n=4 (homozygous *J11* brains); n=2 (wild type brains); n=2 (homozygous *J11* midguts).

Biology Open • Supplementary information

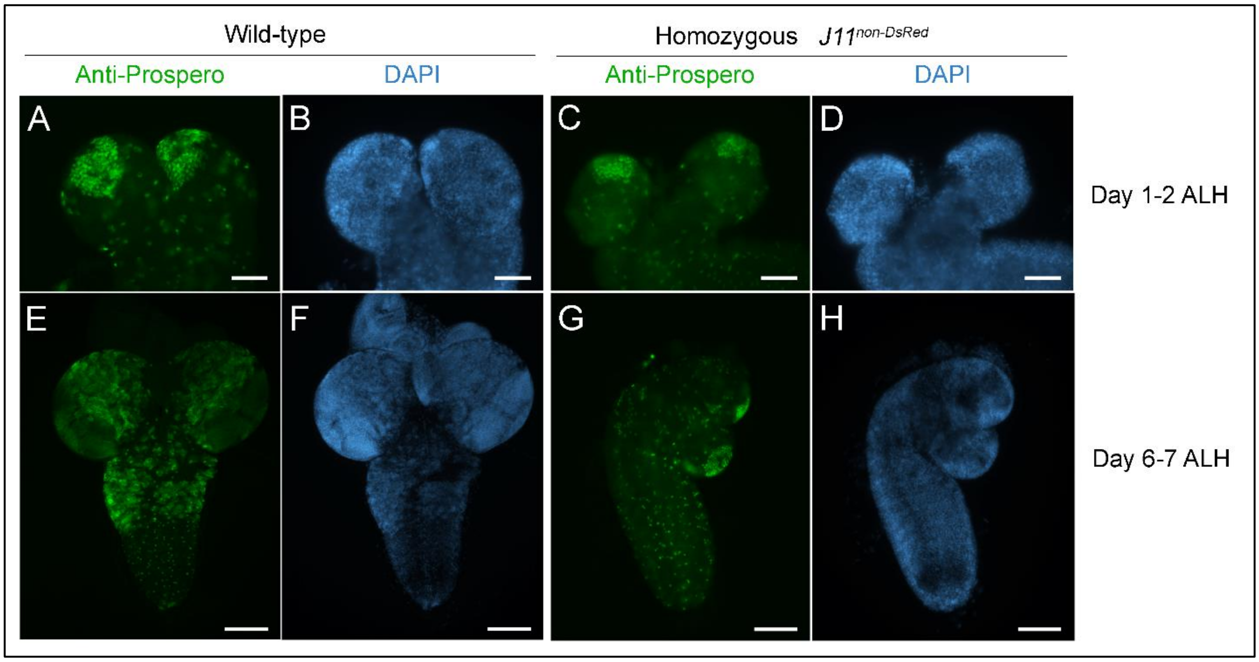

Fig. S3. **Ganglionic mother cell (GMC) populations were significantly reduced upon loss of Nopp140.** Confocal images of wild-type (*w*[1118]; **A**, **B**, **E**, **F**) and homozygous *J11*[non-DsRed] (**C**, **D**, **G**, **H**) larval brains at day 1-2 and 6-7 ALH immuno-stained with anti-prospero (green, prospero is a GMC nuclear marker). n=16 and 17 (wild-type; day 1-2 and 6-7 respectively); n=15 and 18 (homozygous *J11*[non-DsRed]; day 1-2 and 6-7 respectively); >3 technical replicates. Scale bars: 25 µm in a-d, 50 µm in **G** and **H**, 100 µm in **E** and **F**.

Biology Open • Supplementary information

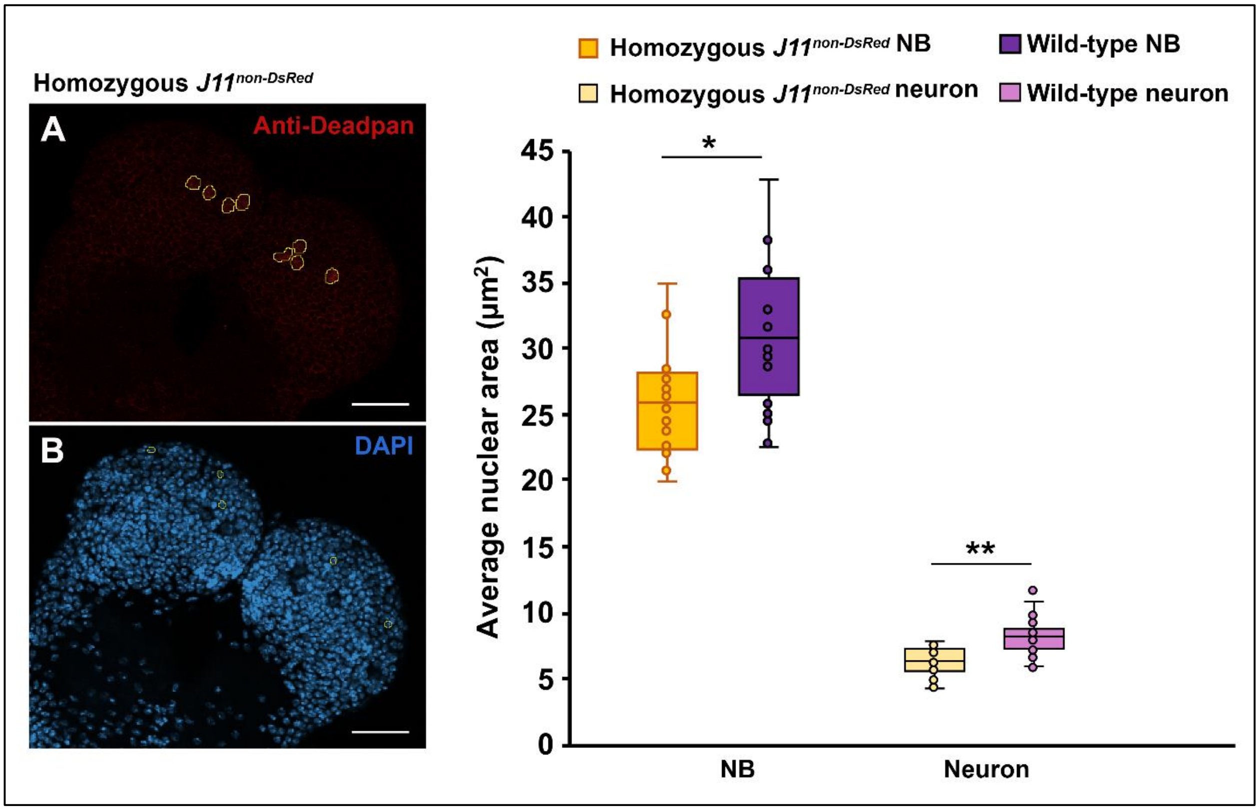

Fig. S4. **The sizes of neuroblasts and neurons are compromised under nucleolar stress.** Neuroblast nuclei marker, Deadpan (Dpn; red), was used to determine nuclear area of neuroblasts, and DAPI (blue) staining in the surrounding neurons was used to determine nuclear area of the neurons. Using the free hand selection tool in Fiji, nuclear outlines were drawn for Dpn-positive neuroblasts (20 nuclei) and DAPI-stained neurons (50 nuclei) on selected 2D confocal images of wild-type and homozygous $J11^{non\text{-}DsRed}$ larval brains at day 2-3 ALH. An example is provided in panels **A** and **B** that show a homozygous $J11^{non\text{-}DsRed}$ larval brain at day 2-3 ALH with nuclear outlines drawn in yellow. Comparisons of the nuclear area of wild-type (neuroblasts: purple, neurons: pink) and homozygous $J11^{non\text{-}DsRed}$ (neuroblasts: orange, neurons: yellow) is provided. Statistical analyses were performed on the raw nuclear area data. Student's t-test: two-tailed with unequal variance, p-values = 0.0015* and 1.60707E-13** Scale bar: 25 µm

Biology Open • Supplementary information

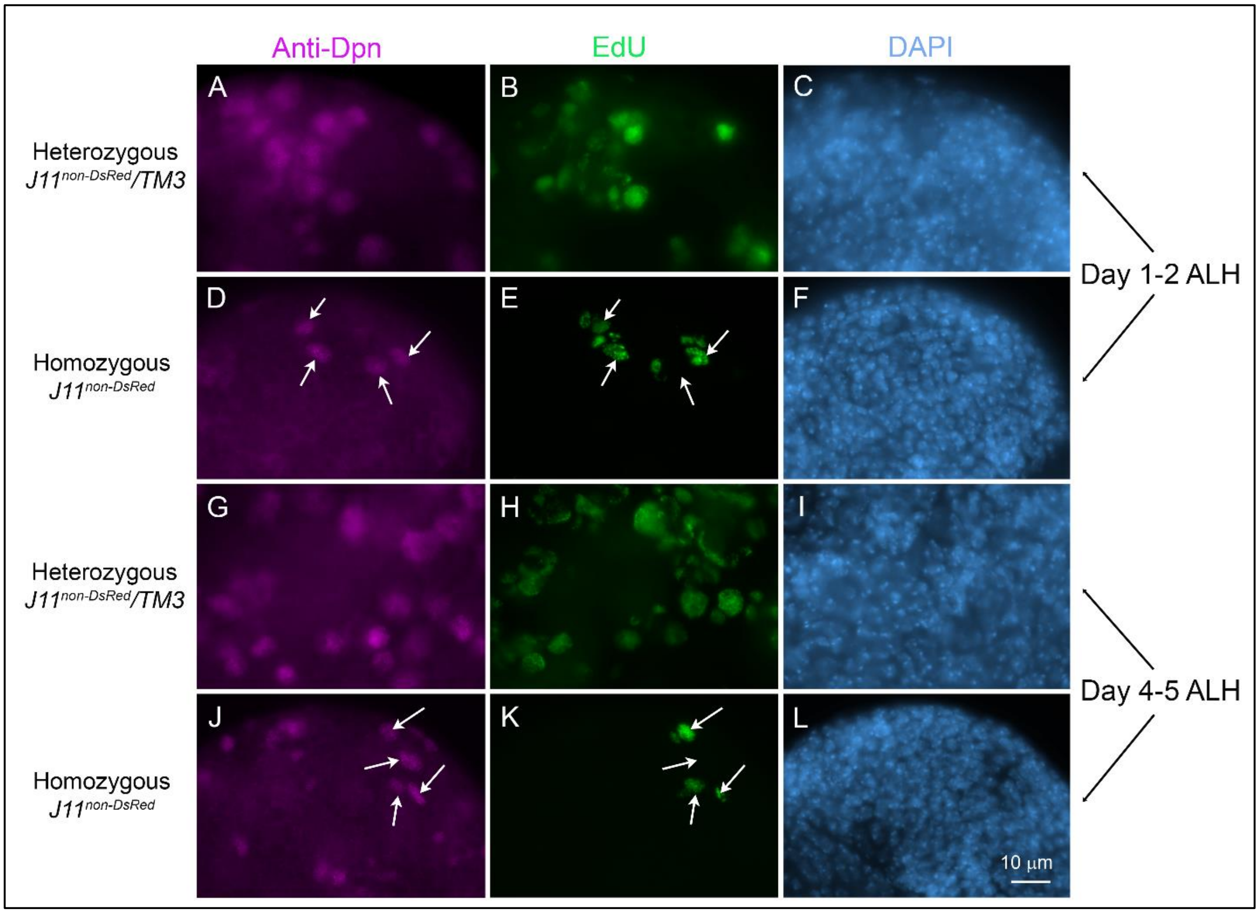

Fig. S5. **Neuroblast proliferation in heterozygous *J11/TM3* larvae relative to homozygous *J11* larvae**. Conventional fluorescence images showing 30 min pulse labeling with EdU (Click-iT Alexa Fluor 488) followed by anti-deadpan (Anti-Dpn) and DAPI labeling. Deadpan is a nuclear marker specific for neuroblasts. Day 1-2 ALH heterozygous brains showed several neuroblasts (panels **A-C**; n=13). Similar to Fig. 6, day 1-2 ALH homozygous *J11* brains contained few neuroblasts; presumably these are the MB NBs (arrows), the majority of which maintained S-phase DNA replication as did their descendent GMCs (panels **D-F** n=5). Day 4-5 ALH heterozygous brains (panels **G-I**; n=23) appeared similar to wild type brains (Fig. 6) in the number of neuroblasts present and engaged in the cell cycle. Day 4-5 ALH homozygous J11 brains still contained few neuroblasts. Presumably only the MB NBs (arrows) versus other apparent neuroblasts were capable of replicating their DNA (panels **J-L**; n=5). Three technical replicates were used for the heterozygous *J11/TM3* labeling.

Biology Open • Supplementary information

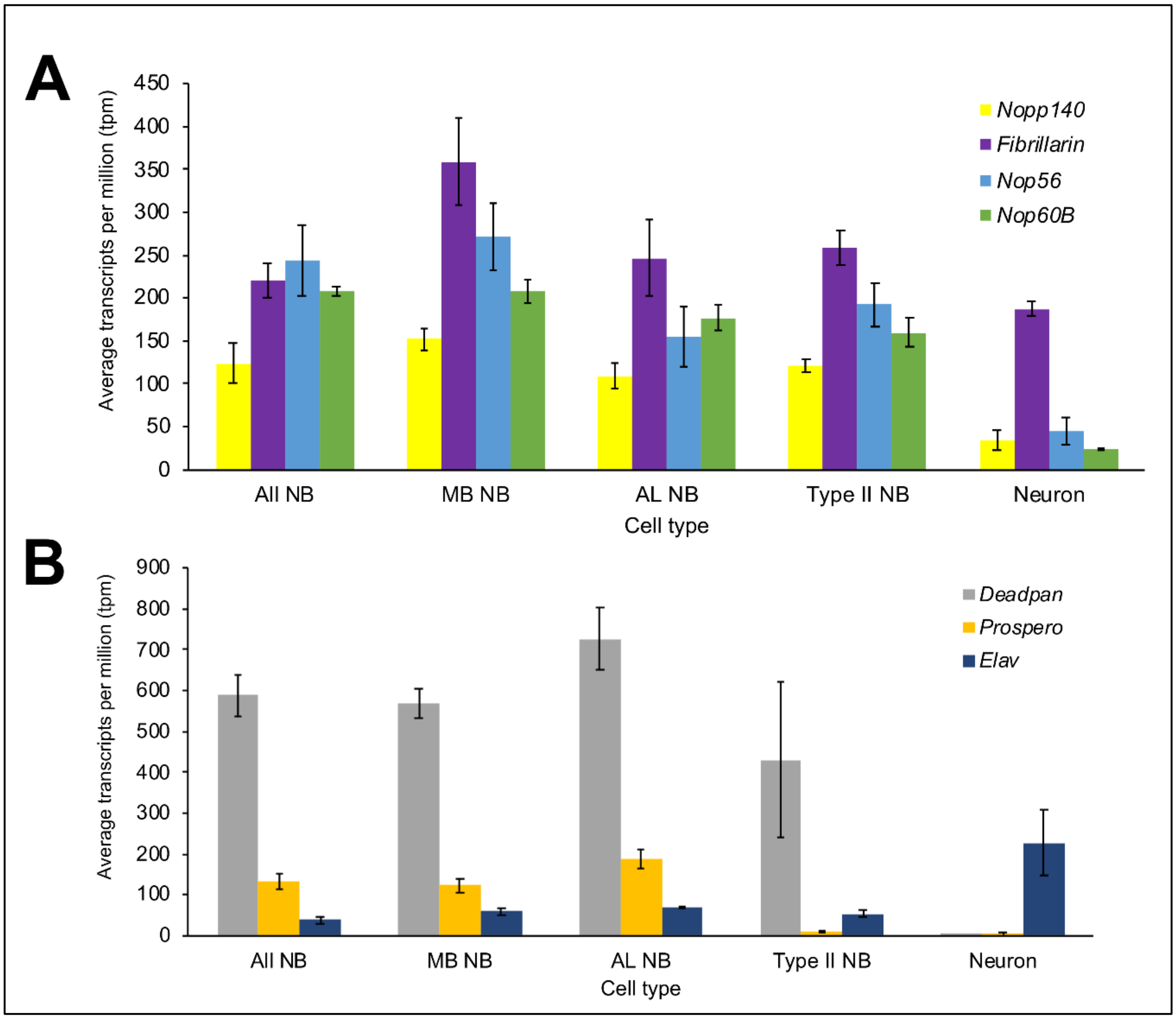

Fig. S6. **Transcriptome analysis of genes encoding ribosome biogenesis factors in lineage-specific *Drosophila* neuroblasts and neurons.** Expression levels of *Nopp140, fibrillarin, Nop56,* and *Nop60B* transcripts (**A**); *Deadpan, Prospero,* and *Elav* (**B**) in *Drosophila* larval NBs and neurons. Transcriptome data obtained from Yang et al. (2016). n=3 (all NB), n=3 (Mushroom Body (MB) NB), n=3 (Antennal Lobe (AL) NB), n=3 (Type II NB), n=2 (neurons).
